# Syndecan-1 Promotes Angiogenesis in Triple-Negative Breast Cancer through the Prognostically Relevant Tissue Factor Pathway and Additional Angiogenic Routes

**DOI:** 10.3390/cancers13102318

**Published:** 2021-05-12

**Authors:** Eyyad Nassar, Nourhan Hassan, Eslam A. El-Ghonaimy, Hebatallah Hassan, Mahmoud Salah Abdullah, Theresa V. Rottke, Ludwig Kiesel, Burkhard Greve, Sherif Abdelaziz Ibrahim, Martin Götte

**Affiliations:** 1 Department of Gynecology and Obstetrics, Münster University Hospital, Albert-Schweitzer-Campus 1, D11, 48149 Münster, Germany; e_nass01@uni-muenster.de (E.N.); n_hass07@uni-muenster.de (N.H.); islamelghonaimy@gmail.com (E.A.E.-G.); Theresa.Rottke@gmx.de (T.V.R.); ludwig.kiesel@ukmuenster.de (L.K.); 2 Biotechnology/Biomolecular Chemistry Program, Faculty of Science, Cairo University, 12613 Giza, Egypt; 201428723@std.sci.cu.edu.eg; 3 Department of Zoology, Faculty of Science, Cairo University, 12613 Giza, Egypt; aheba@sci.cu.edu.eg; 4 Department of Radiotherapy and Radiooncology, University Hospital Münster, 48149 Münster, Germany; greveb@uni-muenster.de

**Keywords:** triple-negative breast cancer, prognosis, Syndecan-1, CD138, angiogenesis, tissue factor, endothelin-1, PAR1, PAR2, 3D co-culture

## Abstract

**Simple Summary:**

Triple-negative breast cancer is an aggressive subtype of breast cancer characterized by tumor angiogenesis and poor patient survival. Here, we analyzed the function of the cell surface molecule Syndecan-1 in tumor angiogenesis in a 3D cell culture system. As a novel finding, we demonstrate that downregulation of Syndecan-1 reduces angiogenesis by decreasing the amount of angiogenesis factors of the tissue factor pathway. Furthermore, we show that the components of this pathway are associated with the prognosis of breast cancer patients. Our study identifies Syndecan-1 and the tissue factor pathway as novel potential therapeutic targets in the aggressive triple-negative subtype of breast cancer, for which no targeted therapies are currently available.

**Abstract:**

Triple-negative breast cancer (TNBC) is characterized by increased angiogenesis, metastasis, and poor survival. Dysregulation of the cell surface heparan sulfate proteoglycan and signaling co-receptor Syndecan-1 is linked to poor prognosis. To study its role in angiogenesis, we silenced Syndecan-1 in TNBC cell lines using a 3D human umbilical vein endothelial cell (HUVEC) co-culture system. Syndecan-1 siRNA depletion in SUM-149, MDA-MB-468, and MDA-MB-231 cells decreased HUVEC tubule network formation. Angiogenesis array revealed reduced VEGF-A and tissue factor (TF) in the Syndecan-1-silenced secretome. qPCR independently confirmed altered expression of *F3*, *F7*, *F2R/PAR1*, *F2RL1/PAR2*, *VEGF-A*, *EDN1*, *IGFBP1*, and *IGFBP2* in SUM-149, MDA-MB-231, and MDA-MB-468 cells. ELISA revealed reduced secreted endothelin-1 (SUM-149, MDA-MB-468) and TF (all cell lines) upon Syndecan-1 depletion, while TF pathway inhibitor treatment impaired angiogenesis. Survival analysis of 3951 patients demonstrated that high expression of *F3* and *F7* are associated with better relapse-free survival, whereas poor survival was observed in TNBC and p53 mutant basal breast cancer (*F3*) and in ER-negative and HER2-positive breast cancer (*F2R, F2RL1*). STRING protein network analysis revealed associations of Syndecan-1 with VEGF-A and IGFBP1, further associated with the TF and ET-1 pathways. Our study suggests that TNBC Syndecan-1 regulates angiogenesis via the TF and additional angiogenic pathways and marks its constituents as novel prognostic markers and therapeutic targets.

## 1. Introduction

Breast cancer is the most frequently diagnosed malignant tumor in women worldwide, with over 1.6 million cases each year. It is the main cause of cancer-related mortality among female patients [1]. Based on the expression of the surrogate receptors, breast cancer can be stratified into different molecular subtypes [2] and this reflects the biological diversity of breast cancer [3]. The hormone receptor-positive molecular subtype represents 64% of the diagnosed breast cancer cases and is characterized by the expression of estrogen receptor (ER) or progesterone receptor (PR) (ER and/or PR) [4]. About 23% of cases are positive for human epidermal growth factor receptor 2 (HER2), of which 32% are hormone receptor-negative and 67% are hormone receptor-positive. The TNBC subtype is ER-, PR-, and HER2-negative and represents only 13% of breast cancer cases [4]; however, it is an aggressive subtype associated with poor outcome. 

Angiogenesis—the formation of new blood vessels from existing vasculature—is a hallmark of cancer and is an essential process for cancer growth and progression [5]. Therefore, interference with the angiogenesis process is of particular relevance as a therapeutic option in cancer. Angiogenesis is a complex process that is highly regulated through a plethora of cues secreted by different tumor microenvironmental cells and multiple signaling pathways, such as vascular endothelial growth factor (VEGF), basic fibroblast growth factor (bFGF), and tissue factor (TF) [3,6]. Notably, the activity of angiogenic factors depends on their interaction with proteoglycan coreceptors at the cell surface [7,8]. 

Syndecan-1 (Sdc-1/CD138), a transmembrane heparan sulfate proteoglycan, is ubiquitously expressed on epithelial cells and functions as a coreceptor for a myriad of chemokines, cytokines, and growth factors [9,10,11]. Sdc-1 is involved in various vital processes relevant to cancer progression, such as cell adhesion, proliferation, migration, invasion, and metastasis [10,11,12,13,14,15]. Of particular relevance to the process of angiogenesis, co-expression of Sdc-1, E-cadherin, and c-Met represents a signature associated with (lymph) angiogenesis-related factors in ductal breast carcinoma in situ [16]. Sdc-1 knockout mice show enhanced corneal angiogenesis mediated by increased leukocyte adhesion [17], whereas Sdc-1 overexpressing mice show abnormal angiogenesis during skin wound repair [18]. A further study showed that treatment with synstatin, a peptide inhibitor that disrupts the interaction of Sdc-1 with αvβ3 and αvβ5 integrins, impairs angiogenesis in vitro and in a mouse model of breast cancer [19]. Sdc-1 is physically associated with VEGFR-2, facilitating VEGF/VEGFR-2 signaling in glomerular endothelial cells [20]. Moreover, in breast cancer, Sdc-1 expression by stromal fibroblasts enhances tumor growth and angiogenesis [21]. We have reported that Sdc-1 is overexpressed and governs the cancer stem cell properties of triple-negative inflammatory breast cancer cells [14]. Interestingly, in the same study, we showed that Sdc-1 downregulation in SUM-149 cells was associated with decreased expression and secretion of the angiogenesis-related factors GRO, IL-6, and IL-8 in 2D models [14]. However, the role of tumor cell-autonomous Sdc-1 expression in the regulation of angiogenesis in the context of tumor-endothelial interactions has not yet been elucidated. Moreover, the role of Sdc-1 in breast cancer angiogenesis has previously not been investigated using unbiased screening approaches. Therefore, we aimed in this study to examine the impact of interference with Sdc-1 expression in TNBC cells on angiogenesis in a 3D co-culture model and to decipher the underlying molecular mechanisms. 

## 2. Materials and Methods 

All supplies and chemicals were from Sigma-Aldrich Chemie GmbH (Taufkirchen, Germany) unless otherwise stated.

### 2.1. Cell Culture

The human TNBC SUM-149 was purchased from BIOIVT (West Sussex, UK) and maintained in Ham’s Nutrient Mixture-F12 (cat. no. N6658) containing 5% FCS (Biochrom GmbH, cat. no. S0615, Berlin, Germany), 1% glutamine, 1% penicillin/streptomycin (cat. no. P433), 5 µg/mL Insulin (cat. no. I9278), and 1 µg/mL Hydrocortisol (cat. no. H4001) and maintained in a humidified atmosphere of 5% CO_2_ at 37 °C. The human MDA-MB-231 and MDA-MB-468 cells were from ATCC/LGC Promochem (Wesel, Germany) and maintained in DMEM containing 1% glutamine (cat. No. D0819), 10% fetal bovine serum, and 1% penicillin/streptomycin in a humidified atmosphere of 7% CO_2_ at 37 °C. HUVECs were purchased from PromoCell (Heidelberg, Germany) [22] and cultured in endothelial cell growth Media MV (EGM) (PromoCell GmbH, cat. no. C-22020, Heidelberg, Germany) at 37 °C and 5% CO_2_. 

### 2.2. siRNA-Mediated Knockdown of Sdc-1 Expression

SUM-149, MDA-MB-231, and MDA-MB-468 cells were transfected with 20 nM silencer pre-validated siRNA (Ambion, cat. no. #s12634 designated as Sdc-1 siRNA#1, targeting exon 2 (NM_002997.4), and #12527 designated as Sdc-1 siRNA#2, targeting exon 5 (NM_002997.4), Cambridgeshire, UK) to silence Sdc-1 or a silencer™ select negative control siRNA (Ambion, cat. no. 4390844) using Dharmafect reagent (Dharmacon™, cat. no. T-2001-03, Colorado, USA) according to the manufacturer’s protocol and as described previously [14,23].

### 2.3. Capillary Tube Formation Assay

A 96-well plate was coated with 50 μL/well Matrigel Matrix (Corning, cat. no. 354230, New York, NY, USA) and allowed to solidify at 37 °C incubator for 30 min. HUVECs (2 × 10^4^/well) with a passage number less than 6 were plated on top of the Matrigel in EGM as a positive control or without EGM as a negative control. Cells were monitored using a confocal time-lapse LSM 880 microscope (Zeiss, Oberkochen, Germany). Tube formation analysis was performed using the “Angiogenesis Analyzer for ImageJ” software [24].

### 2.4. In Vitro 3D Co-Culture System

One hour prior to co-culture, 10,000 of HUVECs and either control and Sdc-1-silenced SUM-149 or MDA-MB-231 cells were maintained in 50 μL Endothelial Basal Medium extract (BEM) (Corning^®^ Matrigel^®^ Basement Membrane Matrix Growth Factor Reduced, cat. no. 354230) and then cells were grown in a 96-well plate as follows: HUVECs were maintained in BEM with and without FCS, control, and Sdc-1 siRNA transfected cells, HUVECs with control, and Sdc-1 siRNA transfected cells. The 96-well plate was maintained for 24 h in a humidified atmosphere of 5% CO_2_ at 37 °C. Cells were stained with Cell Tracker Green CMFDA (Molecular Probes, Thermo Fisher Scientific, cat. no. C2925, Massachusetts, MA, USA) or Cell Tracker Orange CMTMR (Molecular Probes, Thermo Fisher Scientific, cat. no. C2927) according to the manufacturer’s instructions and monitored using confocal time-lapse microscopy (LSM 880), followed by quantitative analysis of tube formation as described above. For TF pathway inhibition, the aforementioned steps were analogously repeated in absence and presence of 50 ng/mL recombinant human CellExpTM tissue factor pathway inhibitor (TFPI), (Sigma-Aldrich Chemie GmbH, cat. no. SRP6458-10 UG).

### 2.5. Proteome Profiler™ Human Angiogenesis Antibody Array

The relative expression levels of 55 angiogenesis-related proteins were detected in the cell culture supernatant using a Proteome Profiler™ Human Angiogenesis Antibody Array according to the manufacturer’s instructions (R&D Systems, cat. no. ARY007, Minneapolis, MN, USA). Briefly, after the 1-h membrane blocking step, cell culture supernatant (200 µL) was pre-incubated for 1 h with biotinylated detection antibodies cocktail (15 µL) and then added to the membrane before incubating overnight at 4 °C on a rocking platform shaker. After a series of washing steps, the membrane was incubated with 2 mL of diluted streptavidin-horseradish peroxidase for 30 min before chemiluminescence detection on a Fusion SL system (PeqLab/VWR, Langenfeld, Germany).

### 2.6. Quantitative Real-Time PCR

After 48 h from siRNA transfection, total cellular RNA was isolated from SUM-149, MDA-MB 231, and MDA-MB-468 cells using innuPREP RNA mini-Kit (Analytik Jena AG, cat. no. 845-KS-2040050, Jena, Germany) and reverse transcribed into cDNA using the High-Capacity cDNA Reverse Transcription Kit (Applied Biosystems, cat. no. 4368814, Foster City, CA, USA). Quantitative real-time PCR was performed in duplicate for each target gene using RT^2^ SYBR Green qPCR Primer Assay (Qiagen, cat. no. 330500, Hilden, Germany) and Takyon™ ROX probe qPCR Kit (Eurogentec GmbH, cat. no. UF-RPMT-B0100, Cologne, Germany) in a 7300 real-time PCR detection system (Applied Biosystems). The relative gene expression levels were assessed using the 2^−ΔΔCt^ method after normalization to the β-actin gene as an internal control. Melting curve analysis was conducted to confirm specific product amplification. The following target genes were assessed: *VEGF-A*, *F3*, *F7*, *IGFBP1*, *IGFBP2*, *EDN1*, *F2R*, and *F2RL1*. Primer sequences are listed in Supplementary Table S1.

### 2.7. ET-1 and Coagulation Factor III/TF ELISA

A total of 2.5 × 10^5^ SUM-149, MDA-MB-231, and MDA-MB-468 cells were seeded in a 6-well plate, then transfected with Sdc-1 siRNA and negative control siRNA. After 24 h, normal medium with FCS was added for only one hour, and the cells were then detached and counted. A total of 1.5 × 10^5^ cells were re-suspended and seeded in a 12-well plate with medium without FCS. After 48 h, the supernatant was collected, centrifuged at 4 °C for 15 min, and used for quantification of ET-1 or coagulation factor III/TF by ELISA (Quantikine ELISA endothelin-1 and coagulation factor III/TF kits, R&D Systems, cat. no. DET-100, and DCF300, respectively). 

### 2.8. Survival and Expression Analysis

Using the online tool kmplot.com [25], prognostic analysis for angiogenesis-related genes was performed in correlation with the relapse-free survival of 3951 breast cancer patients (see [25] for details on patient data). The median was used as a cut-off value to differentiate between high and low expression and to produce a strong correlation between different categories by reducing the influence of the outliers. The Jet-Set probe set was selected as the recommended ideal probe set for each target gene. Breast cancer patients were molecularly classified based on the expression of the surrogate markers and clinical features, namely ER, PR, and HER2 status, lymph node status, the grade of the tumor, intrinsic subtype, and p53 status. The Affymetrix IDs are 204363_at (F3) 204363_at (F7), 203989_x_at (F2R); 213506_at (F2RL1), and 210512_s_at (VEGF-A). 

### 2.9. Protein-Protein Interaction Analysis

STRING (Search Tool for the Retrieval of Interacting Genes/Proteins) (version 11) (http://string-db.org/) (accessed on 29 January 2021) [26] was used to create an in silico protein interaction network for Sdc-1 in relation to the TF pathway signaling-related products; F3, F7, F2R, and F2RL1 in addition to VEGF-A, EDN1, and insulin-like growth factor (IGF) signaling pathways. For the enrichment analysis, STRING implements well-known classification systems such as Gene Ontology (GO) and KEGG (Kyoto Encyclopedia of Genes and Genomes). 

### 2.10. Statistical Analyses

GraphPad Prism 4.02 (GraphPad Software, La Jolla, CA, USA) was used for the statistical analysis of experimental assays, and Student’s *t*-test was used to assess the significant differences between the two groups. For survival analysis, in the R statistical environment, we utilized the Kaplan–Meier Plotter database via the statistical package “survival” to calculate Kaplan–Meier survival curves and the number-at-risk. Furthermore, the hazard ratio (and 95% confidence intervals) and log-rank *p* were calculated for each gene [27]. The statistical analysis was considered significant when *p* < 0.05.

## 3. Results

### 3.1. Sdc-1 Silencing in TNBC Cells Impairs Capillary-Like Tube Formation in a HUVEC Co-Culture Model

**Figure 1:**
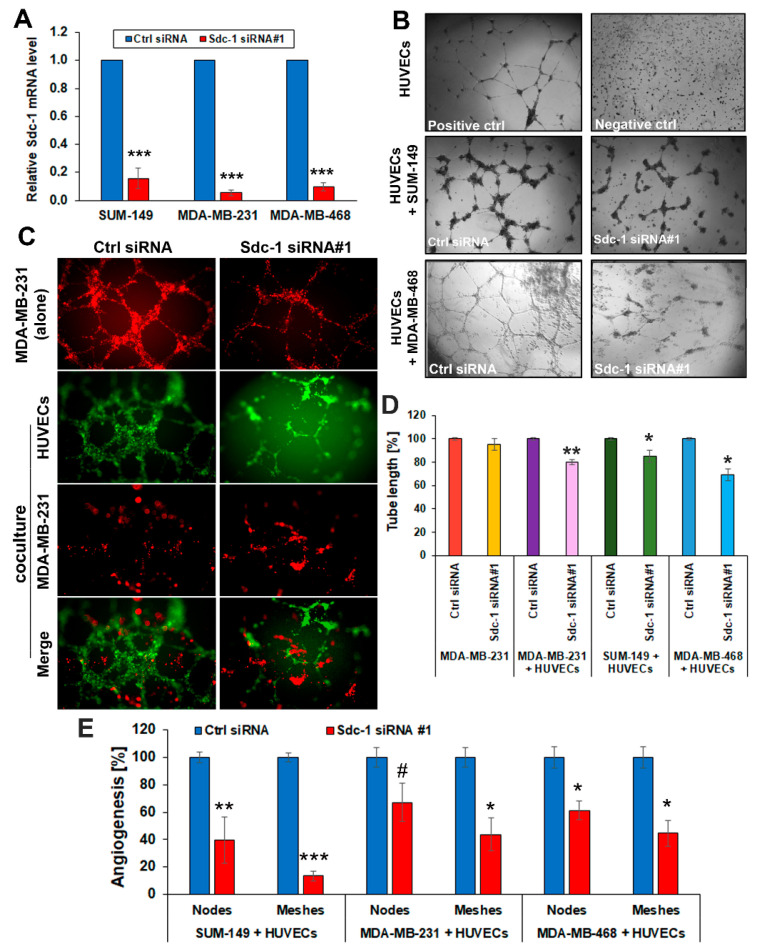
Sdc-1 depletion in TNBC cells restrains angiogenesis network formation of HUVECs. (**A**) Sdc-1 knockdown was confirmed by qPCR in the triple-negative cell lines SUM-149, MDA-MB-231, and MDA-MB-468. (**B**–**E**) 3D co-culture models of HUVECs and the control siRNA and Sdc-1 siRNA transfected SUM-149, MDA-MB-468, and MDA-MB-231 cells. (**B**) Phase-contrast images for HUVECs were either grown in 3D alone (as negative or positive controls) or co-cultured with control and Sdc-1-suppressed SUM-149 and MDA-MB-468 cells for 24 h. (**C**) Confocal immunofluorescence microscopy shows tubule formation by only HUVECs (green fluorescent staining) and not by MDA-MB-231 cells (red fluorescent staining) in a co-culture 3D system. Notably, MDA-MB-231 cells formed tubules when cultured alone in 3D on Matrigel. (**D**) Quantitative analysis of HUVEC tubulogenesis, namely the total length of HUVEC tubules. (**E**) Quantitative analysis of nodes and meshes formed by HUVECs as analyzed by angiogenesis analyzer software. Panels (**A**,**D**,**E**): Data represent the mean ± SEM, *n* = 3. *** *p* < 0.001, ** *p* < 0.01, * *p* < 0.05, and # *p* = 0.09 as determined by Student’s *t*-test.

Sdc-1 has an influence on a myriad of biological processes, relevant to tumor progression [28]; however, the role of cell-autonomous Sdc-1 expression in TNBC cells on in vitro angiogenesis has not been explored. Therefore, we employed an in vitro 3D co-culture system comprising HUVECs and TNBC cells. After the successful confirmation of siRNA-mediated Sdc-1 silencing by ~80% using qPCR (Figure 1A, Supplementary Figure S1A), control and Sdc-1-silenced TNBC cells were co-cultured with HUVECs and the tube network formation was monitored under 3D co-culture conditions. Our data indicate decreased angiogenesis evident by the reduced HUVEC tube formation when HUVECs were co-cultured with MDA-MB-468 (Figure 1B), SUM-149 (Figure 1B, Supplementary Figure S1B), and MDA-MB-231 cells (Figure 1C) subjected to Sdc-1 depletion, in comparison with those co-cultured with control cells assessed by phase-contrast and confocal immunofluorescence microscopy (Figure 1B–D). The length of tubes formed by HUVECs was significantly suppressed upon 3D co-culturing with both Sdc-1 knockdown SUM-149 (*p* < 0.05), MDA-MB-468 (*p* < 0.05), and MDA-MB-231 (*p* < 0.01) cells relative to control transfectants (Figure 1D). Furthermore, a significant reduction of the number of meshes of HUVECs was observed when co-cultured with Sdc-1-depleted SUM-149 (*p* < 0.001), MDA-MB-231 (*p* < 0.05), and MDA-MB-468 (*p* < 0.05) cells, and of the number of nodes in SUM-149 (*p* < 0.01) and MDA-MB-468 (*p* < 0.05) cells, with a trend for reduction seen in MDA-MB-231 cells (*p* = 0.09) (Figure 1E, Supplementary Figure S1). Similar findings of reduced angiogenesis of HUVECs were obtained upon 3D co-culturing with Sdc-1-depleted SUM-149 and MDA-MB-231 cells using siRNA#2, targeting a different exon of Sdc-1 (Supplementary Figure S1C–E). To exclude the possibility of tube formation by SUM-149 or MDA-MB-231 cells, HUVECs were stained with either cell tracker green or red fluorescent dye. As depicted in Figure 1C and Supplementary Figure S1B, confocal immunofluorescence microscopy demonstrated that angiogenesis tubes in co-culture were exclusively formed by HUVECs. However, we observed that MDA-MB-231 cells were able to form tubules when cultured alone in 3D on Matrigel, and that tube formation was reduced upon Sdc-1 silencing and abolished when the cells were co-cultured with HUVECs (Figure 1C,D).

### 3.2. Sdc-1 Knockdown Influences Expression and Secretion of Angiogenesis-Related Factors in SUM-149, MDA-MB-231, and MDA-MB-468 cells

**Figure 2:**
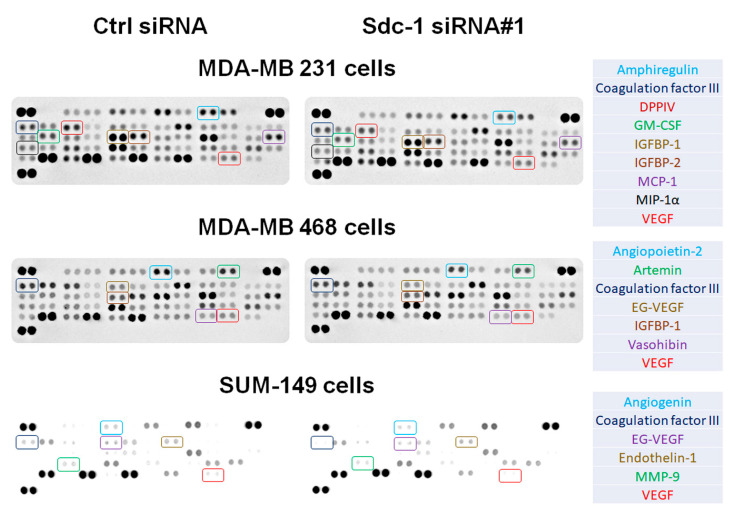
Sdc-1 expression affects the expression and secretion of angiogenesis-related factors. Profiling of angiogenesis-regulating molecules secreted by control and Sdc-1 siRNA transfected cells in a 3D co-culture model with HUVECs as analyzed by proteome profiler™ human angiogenesis antibody array. Media conditioned by the secretome of control or the indicated Sdc-1 siRNA-treated TNBC cells co-cultured in 3D with HUVECs were collected and subjected to profiling. Colored boxes indicate common and cell type-specific dysregulated factors. See Supplementary Figure S2 for densitometric quantification.

**Figure 3:**
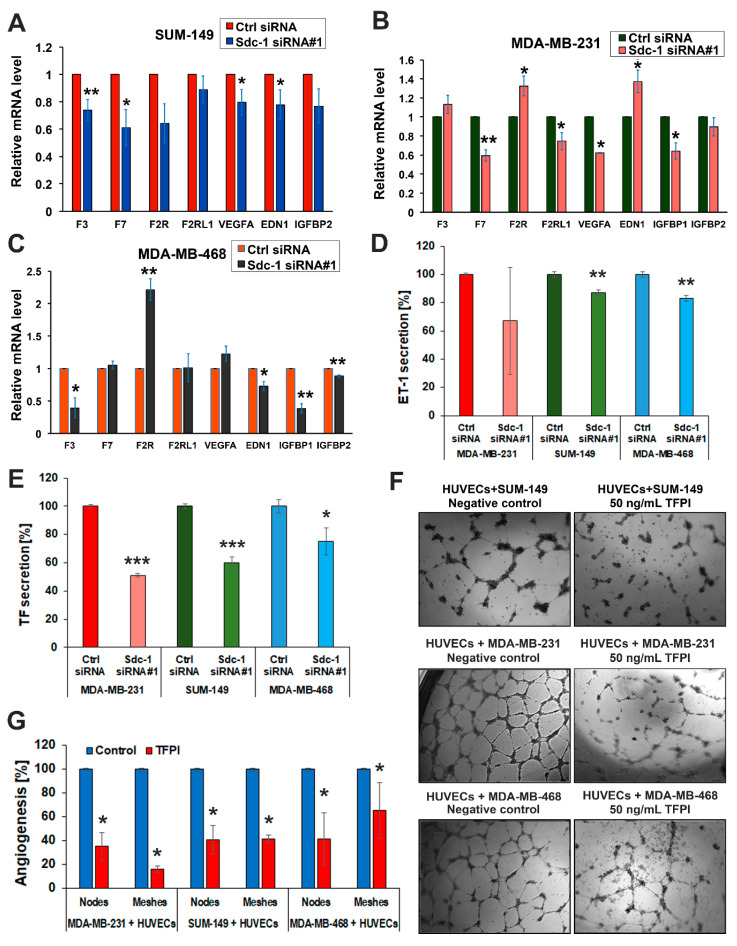
Impact of Sdc-1 on the gene expression of angiogenic factors and functional impact of tissue factor on angiogenesis. (**A**–**C**) Sdc-1 silencing differentially regulates expression of the TF signaling pathway and angiogenic factors in SUM-149, MDA-MB-231, and MDA-MB-468 cells as assessed by qPCR. (**D**) Sdc-1 knockdown decreases EDN1 secretion in SUM-149 and MDA-MB-468 cells. EDN1 secretion was quantified by ELISA in the cell culture supernatants of control and Sdc-1 knockdown SUM-149, MDA-MB-231, and MDA-MB-468 cells collected 48 h post-transfection. (**E**) Sdc-1 knockdown decreases coagulation factor III/TF (F3) secretion in TNBC cells. Coagulation factor III/TF secretion was quantified by ELISA in the cell culture supernatants of control and Sdc-1 knockdown SUM-149, MDA-MB-468, and MDA-MB-231 cells collected 48 h post-transfection. (**F**) Representative images of the in vitro 3D co-culture model of HUVECs with TNBC cells treated with 50 ng/mL tissue factor pathway inhibitor (TFPI). TFPI treatment blunts the ability of tubule formation of HUVECs in co-culture with TNBC cells. (**G**) Quantitative analysis of nodes and meshes formed by HUVECs in coculture with TNBC cells as analyzed by angiogenesis analyzer software. Data represent the mean ± SEM, n ≥ 3. *** *p* < 0.001, ** *p* < 0.01, and * *p* < 0.05 as determined by Student’s *t*-test.

To identify a possible Sdc-1-dependent regulation of angiogenic factors, we used an unbiased array (proteome profiler for angiogenesis factors) to simultaneously detect 55 angiogenesis-related proteins. VEGF-A and Coagulation Factor III (TF/F3) proteins were blunted in the secretome of Sdc-1-depleted cells relative to controls (Figure 2, Supplementary Figure S2 and Table S2). In addition, several angiogenic factors were downregulated in a cell type-specific manner in Sdc-1-depleted SUM-149, MDA-MB-231, and MDA-MB-468 cells (Supplementary Figure S2). We next independently analyzed the expression of angiogenesis-related factors at the mRNA level by qPCR. Sdc-1 silencing in SUM-149 cells resulted in a significant downregulation of *F3* encoding coagulation Factor III/TF (*p* < 0.01), *F7* encoding coagulation factor VII (*p* < 0.05), *VEGF-A* (*p* < 0.05), and *EDN1* encoding endothelin-1 (ET-1) (*p* < 0.05) mRNA expression levels (Figure 3A). Furthermore, a significant reduction of *F7* (*p* < 0.01), *F2RL1 (PAR2)* encoding F2R like trypsin receptor 1 (*p* < 0.05), *VEGF-A* (*p* < 0.05), and insulin-like growth factor-binding protein-1 (*IGFBP1*) mRNA expression levels (*p* < 0.05), and a significant upregulation of *F2R (PAR1)*, encoding coagulation factor II thrombin receptor, and *EDN1* mRNA levels were detected upon Sdc-1 silencing in MDA-MB-231 cells (Figure 3B). In Sdc-1-depleted MDA-MB-468 cells, *F3* (*p* < 0.05), *EDN1* (*p* < 0.05), *IGFBP1* (*p* < 0.01), and *IGFBP2* (*p* < 0.01) mRNA levels were lower, whereas *F2R* mRNA expression levels were higher (*p* < 0.01) than in control cells (Figure 3C). Next, we confirmed the effect of Sdc-1 knockdown on the secretion of ET-1. Sdc-1 silencing significantly reduced the secreted ET-1 in media conditioned by SUM-149 and MDA-MB-468 when compared with control cells (both *p* < 0.01, Figure 3D) as quantified by ELISA. ET-1 secretion was not significantly altered in MDA-MB-231 cells. We further assessed the protein levels of coagulation Factor III/TF in serum-starved media collected from control and Sdc-1 silenced TNBC cells. ELISA results show a significant decrease of coagulation Factor III/TF by ~40% in SUM-149 and MDA-MB-231 cells (both *p* < 0.001), and by about 25% in MDA-MB-468 cells (*p* < 0.05) upon Sdc-1 silencing (Figure 3E). These findings were verified in SUM-149 and MDA-MB-231 cells using Sdc-1 siRNA#2, and similar findings were confirmed (Supplementary Figure S1F,G). Finally, we substantiated the involvement of the TF pathway in regulating angiogenesis, where all TNBC cell lines in a 3D co-culture system with HUVECs were treated with 50 ng/mL TFPI. The tube formation ability of HUVECs was significantly inhibited, implying the role of TF pathway in angiogenesis induction (Figure 3F,G). Together, these data suggest that TNBC-autonomous Sdc-1 expression affects the angiogenic potential of HUVECs via regulation of different components of TF pathway and additional angiogenic factors. 

### 3.3. Expression of Coagulation Factor III/TF Is Associated with Worse Relapse-Free Survival in Breast Cancer Patients with Different Classifications

**Figure 4:**
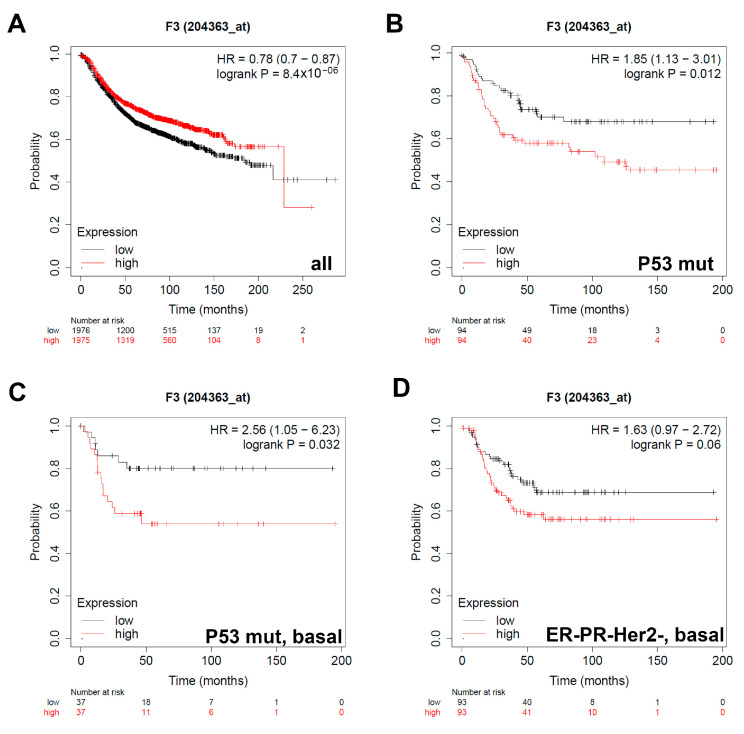
The prognostic value of the expression of F3 in patients with breast cancer. Kaplan–Meier curves are plotted based on the expression of F3 in (**A**) all patients (*n* = 3951), (**B**) p53 mutated (*n* = 1149), (**C**) patients with the intrinsic molecular classification Basal and p53 mutated (*n* = 901), and (**D**) Triple-negative tumors of the basal subtype (*n* = 186). Curves were analyzed using the log-rank test. Log-rank *p* values and hazard ratios are shown. Log-rank *p* values and hazard ratios (HRs; 95% confidence interval in parentheses) are shown. The corresponding Affymetrix ID is 204363_at.

Sdc-1 knockdown affected the expression of different angiogenic factors including the TF pathway constituents’ coagulation factor III/TF, Factor VII/F7, F2R/PAR1 and F2RL1/PAR2, and the key angiogenic factor VEGF-A. To assess the prognostic value of the Sdc-1-dependent factors, we correlated their expression with the relapse-free survival (RFS) for 3951 breast cancer patients using the online tool Kaplan–Meier Plotter as we described before [29]. RFS was determined in the whole collective, and in subgroups of patients stratified according to the expression of hormonal receptors (ER and PR) and HER2, intrinsic subtypes, lymph node status, grade status (I-III), and p53 status (wild-type vs. mutant). In agreement with several previous studies establishing a prognostic value for VEGF-A in breast cancer [30,31], a high VEGF-A expression was associated with poor RFS in all patients (*n* = 3951), ER-patients (*n* = 801), PR- patients (*n* = 549), patients with HER2- status (*n* = 800), triple-negative patients (*n* = 255), and patients with grade II tumors (*n* = 901) (Supplementary Table S3). Regarding the TF pathway, in the collective of all 3951 patients, high F3 expression was associated with longer RFS (HR = 0.78, *p* = 8.4 × 10^−6^) (Figure 4A). In contrast, high F3 expression was correlated with shorter RFS, when patients were stratified according to p53 mutation status (HR = 1.85, *p* = 0.012, Figure 4B) and to p53 mutation with a basal subtype (HR = 2.56, *p* = 0.032, Figure 4C). For all other stratifications, we did not observe any prognostic value for F3 expression (results not shown). In addition, a trend for a poor survival with high F3 expression was seen in the subgroup of triple-negative tumors of the basal subtype (HR = 1.63, *p* = 0.06, Figure 4D) (Table 1). 

### 3.4. Expression of F7/Factor VII Is Associated with Better Relapse-Free Survival and Expression of F2R/PAR1 and F2RL1/PAR2 with Worse Relapse-Free Survival in Breast Cancer Patients with Different Classifications

**Figure 5:**
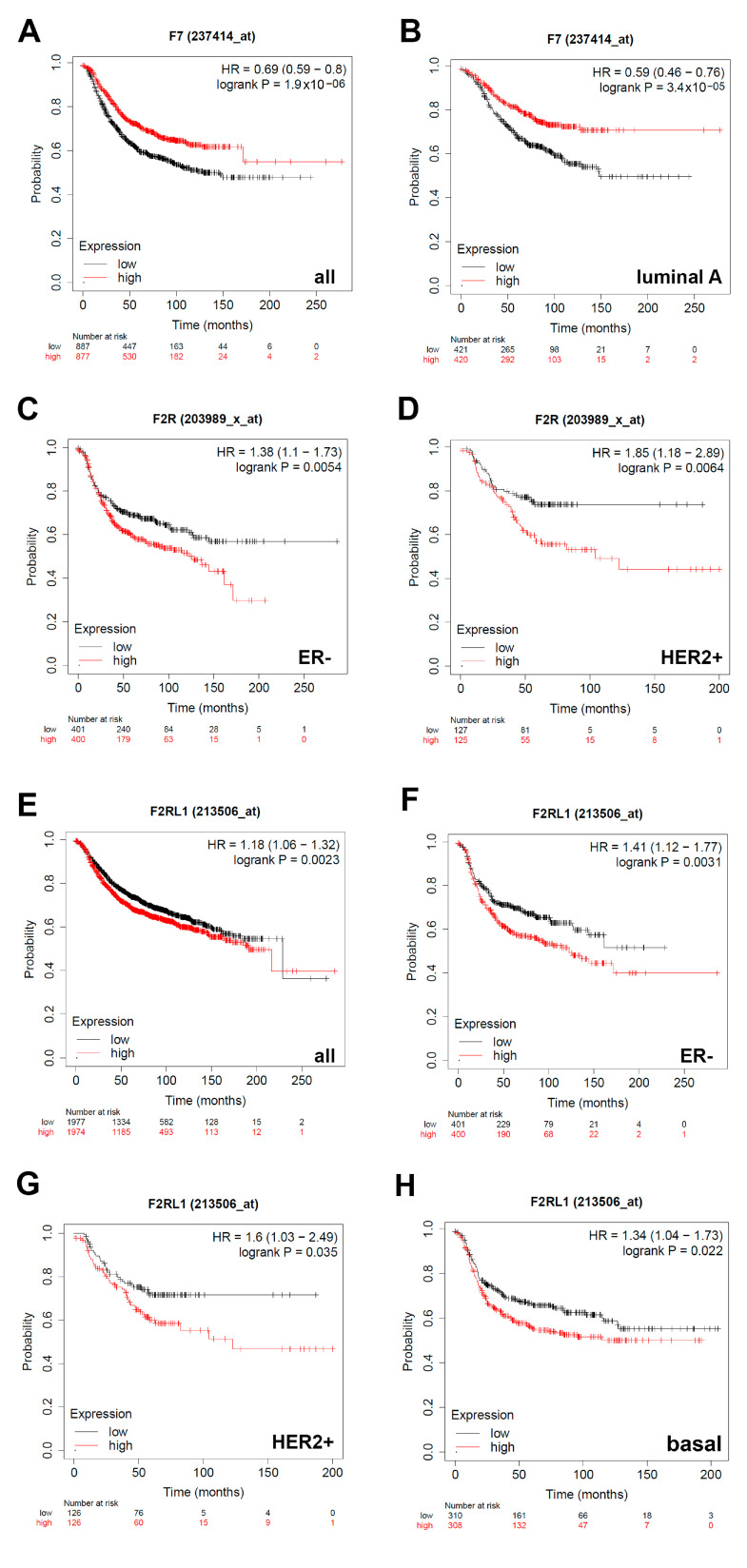
The prognostic value of the expression of F7 (factor VII), F2R (PAR1), and F2RL1 (PAR2) in patients with breast cancer. Kaplan–Meier curves are plotted based on expression of F7 in (**A**) all patients showing F7 expression (*n* = 1734), and (**B**) patients with luminal A classification (*n* = 841). Expression of F2R in (**C**) ER-classified patients (*n* = 801) and (**D**) HER2 + classified patients (*n* = 252). Expression of F2RL1 in (**E**) all patients (*n* = 3951), (**F**) ER- classified patients (*n* = 801), (**G**) HER2 + classified patients (*n* = 252), and (**H**) patients with tumors of the basal subtype (*n* = 618). Curves were analyzed using the log-rank test. Log-rank *p*-values and hazard ratios (HRs; 95% confidence interval in parentheses) are shown. The corresponding Affymetrix IDs are: F7: 204363_at; F2R: 203989_x_at; F2RL1: 213506_at.

We next aimed at establishing a prognostic value for the additional TF pathway constituents that were expressed in a Sdc-1-dependent manner in our study employing KM Plotter survival analysis. A high expression of factor VII/F7 was associated with improved RFS in the whole collective of all 3951 patients (HR = 0.69, *p* = 1.9 × 10^−6^) (Figure 5A) and patients with luminal A tumors (HR = 0.59, *p* = 3.4 × 10^−5^) (Figure 5B). In contrast, a high expression of F2R/PAR1 and F2RL1/PAR2 was associated with a decreased RFS in breast cancer patients of different subtypes. A high F2R expression was associated with poor prognosis in the ER- (HR 1.38, *p* = 0.0054) (Figure 5C) and HER2 + (HR1.85, *p* = 0.031) (Figure 5D) classifications, whereas high expression of F2RL1/PAR2 was associated with a worse outcome independent of the subtype (HR1.18, *p* = 0.0023) (Figure 5E), in the ER- (HR1.41, *p* = 0.0031) (Figure 5F) and HER2 + (HR1.6, *p* = 0.035) (Figure 5G) classification, as well as the basal subtype (HR1.34, *p* = 0.022) (Figure 5H). Overall, these data highlight the prognostic relevance of the Sdc-1-dependent angiogenic pathways identified in this study. To assess if the differential prognostic value of the Sdc-1-dependent angiogenic factors was associated with altered expression patterns in well-characterized human breast cancer model cell lines, we compared the expression of the factors analyzed in this study in a panel of 33 cell lines using RNASeq data of the EMBL-EBI expression atlas (release 37 March 2021) [32] (Supplementary Figure S3). Apart from the observation of a low expression of IGFBP1 and F7 in many cell lines, no clear subtype-assignment of angiogenic factor expression could be made based on RNA Seq data. Importantly, F3 and VEGFA were strongly expressed across the cell line panel.

### 3.5. Protein Network Analysis of Sdc-1 and Angiogenesis-Related Proteins

**Figure 6:**
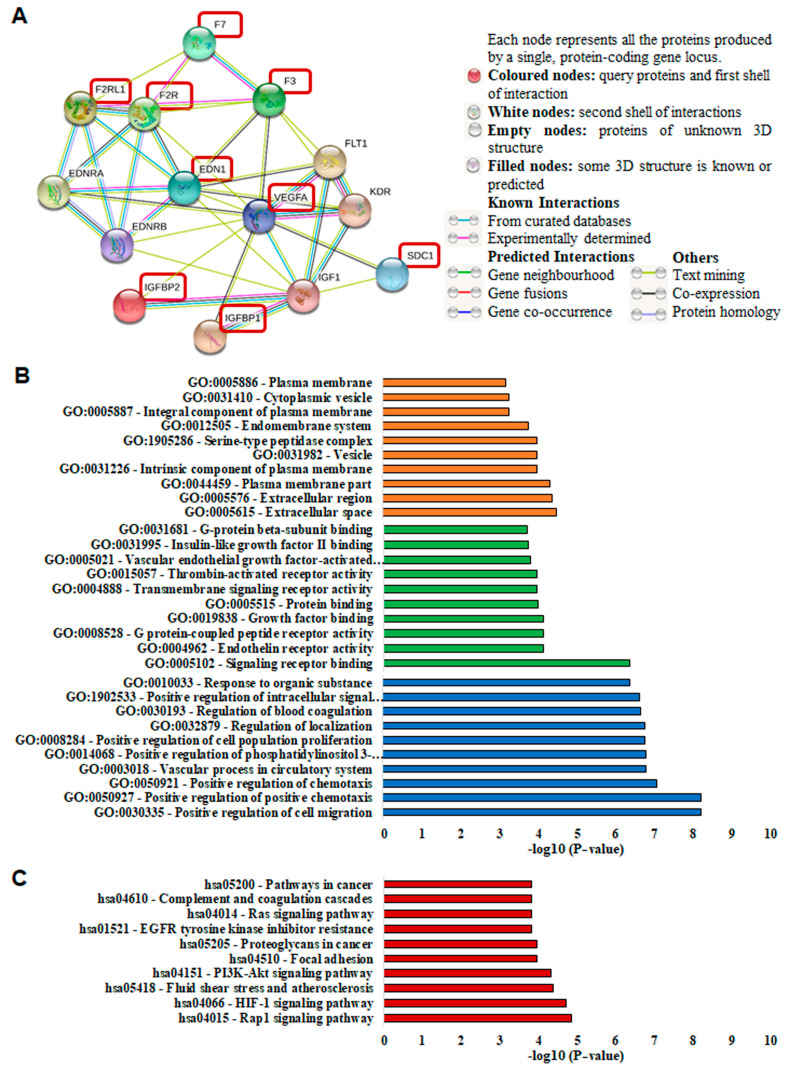
The protein–protein interaction network of Sdc-1 with F3, F7, VEGF-A, EDN1, F2R, F2RL1, IGFBP1, and IGFBP2. (**A**) Interaction analysis of Sdc-1 with F3, F7, VEGF-A, EDN1, F2R, F2RL1, IGFBP1, and IGFBP2 was performed using the STRING database (http://string-db.org/) (accessed on 29 January 2021). The proteins of interest are highlighted in dark red boxes. (**B**) Gene ontology (GO) analysis of Sdc-1, EDN1, F3, F7, VEGF-A, F2R, and F2RL1. The most significant GO terms (*p* < 0.05) in the cellular component (orange), molecular functions (green), and biological processes (blue) branches are shown. (**C**) KEGG pathway analysis. All the selected significant values are presented in (−log10) transformation.

STRING network analysis was used to reveal the biological interactions between Sdc-1 and the angiogenesis-related factors, namely F3, F7, VEGF-A, EDN1, F2R, F2RL1, IGFBP1, and IGFBP2 [26]. The analysis showed that there are close interactions between Sdc-1 and VEGF-A and that Sdc-1 interacts with IGF1. VEGF-A, in turn, has close interactions with EDN1, F3, F2R, IGF1, IGFBP1, and IGFBP2. As shown in Figure 6A, F3, F2R, F7, and F2RL1 are highly interconnected and there is a strong interaction between EDN1, F2R, and F2RL1. We analyzed GO enrichment to evaluate the cellular component (orange), molecular functions (green), and biological processes (blue) related to the proteins of interest. The most significant GO terms (*p* < 0.05) in each classification are presented in Figure 6B and Supplementary Table S4. KEGG pathway analysis (red) is shown in Figure 6C and Supplementary Table S5. Remarkably, Rap1, PI3K-Akt, HIF-1, focal adhesion, Ras, and VEGF signaling pathways, regulation of actin cytoskeleton, and proteoglycans in cancer were found to be linked to the proteins of interest (Figure 6C and Supplementary Table S5). Overall, the STRING analysis confirms the interconnection of the Sdc-1-dependent factors identified in this study.

## 4. Discussion

In this study, we employed an in vivo-inspired 3D co-culture model of Sdc-1-depleted TNBC cells and HUVECs to study the impact of tumor cell Sdc-1 on angiogenesis in a defined setting. Sdc-1 depletion in the TNBC cells reduced capillary tubule-like formation by HUVECs and suppressed expression and secretion of angiogenesis-related factors. An important and novel finding of this study is that TF pathway components governing angiogenesis of HUVECs are prominently regulated by Sdc-1 expression on TNBC cells. It has been shown that MDA-MB-231 and MDA-MB-468 TNBC cells, as well as clinical TNBC tissue samples, express TF, and RNASeq data support this finding (Supplementary Figure S3) [33]. Given the lack of targeted therapies for TNBC, TF emerges as a promising target. Notably, our KM Plotter survival analysis establishes a prognostic value for several constituents of the TF pathway in breast cancer, emphasizing the clinical relevance of our findings.

Sdc-1 acts as a co-receptor for several angiogenic and growth factors [9,11,13,34]. We and others have shown that Sdc-1 is a modulator of angiogenesis in preclinical and clinical settings. For example, Sdc-1-deficient mice featured increased corneal angiogenesis [18] and delayed dermal wound repair caused by the soluble Sdc-1 ectodomains was observed in mice overexpressing Sdc-1 [18]. Mechanistically, altered recruitment of leukocytes secreting proangiogenic cytokines and an altered proteolytic milieu contributed to the Sdc-1-dependent angiogenesis phenotype in vivo. In breast cancer, Sdc-1 along with E-cadherin and c-met constitutes an expression signature associated with angiogenic and lymphangiogenic factors in ductal carcinoma in situ [16]. Moreover, Sdc-1 of stromal fibroblasts induced breast carcinoma growth and angiogenesis in vivo [21]. Clinically, the same study confirmed this finding in primary human breast cancer specimen, where stromal Sdc-1 expression was significantly associated with tumor vascularity (i.e., both vessel density and total vessel area). The regulatory role of Sdc-1 in angiogenesis network formation was also assigned in another study, where Sdc-1 was shown to be a prerequisite for the activation of αvβ3 and αvβ5 integrins during angiogenesis and breast tumorigenesis in vivo [19,35]. These data suggest that Sdc-1 is able to modulate angiogenesis via several different pathways, which is in accordance with its pleiotropic role in physiology and tumor progression [36]. Apart from the central angiogenesis regulator VEGF-A, our study extends these findings to the tissue factor pathway, which apparently plays an important Sdc-1-dependent role in the communication of TNBC and endothelial cells during angiogenesis. 

Angiogenesis is a complex process regulated by multiple molecular factors. VEGF-A is a key player regulating angiogenesis of different human malignancies, including breast cancer [37]. Our qPCR data show downregulation of VEGF-A mRNA levels in SUM-149 and MDA-MB-231 cells. Furthermore, relative to control cells, the angiogenesis array analysis revealed that the secretome of Sdc-1-silenced TNBC cells contained lower VEGF-A, which may explain the observed inhibition of in vitro 3D capillary tube formation. This is in agreement with the notion that Sdc-1 knockdown myeloma cells formed fewer and smaller tumors exhibiting diminished levels of VEGF and impaired development of blood vessels in mice [38]. Moreover, Sdc-1 facilitates angiogenesis of multiple myeloma endothelial cells by modulating VEGF/VEGFR-2 signaling [39], providing an additional mechanistic link.

Other important factors regulating angiogenesis are TF, F7, and the protease-activated receptors (F2R/PAR-1 and F2RL1/PAR-2) [40,41]. The local and systemic activation of the coagulation cascade evoked by tumor TF represents the main cause of cancer-associated thrombosis [42], a feature associated with breast cancer [43]. ELISA data revealed significantly decreased secretion of TF in both SUM-149 and MDA-MB-231 cells following Sdc-1 siRNA knockdown. Moreover, significantly downregulated F3 mRNA levels were detected in Sdc-1-depleted SUM-149 and MDA-MB-468 cells. Additionally, our pharmacological inhibition experiments of TF pathway indicate that SUM-149 cells promote capillary tube formation and angiogenesis via the TF pathway. The non-enzymatic activity of heparanase—known to be modulated by Sdc-1 expression—induces F3 expression in cancer cells and impacts cancer progression [44,45]. We have recently discovered an overexpression of heparanase and Sdc-1 in triple-negative inflammatory breast cancer, an aggressive form of breast cancer represented by the SUM-149 cell line employed in this study [14,46]. F3 promotes angiogenesis of the microvascular endothelial cells [47] and the overexpression of tumor F3-induced increased angiogenesis enables human melanoma xenografts to form lung metastasis [48]. Similar to Sdc-1 mode of action, spliced F3 binds to the endothelial cell integrin αvβ3 and α6β1 to evoke tumor angiogenesis via protein tyrosine kinase-2 signaling [49]. Notably, full-length F3 induces tumor angiogenesis via inducing the expression of angiogenesis-related factors IL-8, CXCL-1, and VEGF-A [50]. Consistently, we have previously shown that IL-8 was downregulated upon Sdc-1 silencing in SUM-149 cells [14]. TF expression is essentially regulated by different signaling pathways, including EGF/EGFR, HGF/c-Met, TGF-β/TGF-βR, and VEGF/VEGFR [51]. Expression of these ligands and/or their receptors besides their downstream effectors have been demonstrated to be regulated by Sdc-1 expression. For example, we have shown that EGFR expression was repressed in SUM-149 cells when subjected to Sdc-1 depletion [14]. Of note, co-expression of Sdc-1 and c-Met represents a signature associated with angiogenic and lymphangiogenic-related factors in ductal breast carcinoma in situ [16]. Taken together, it would be conceivable that a Sdc-1/F3/VEGF-A axis may exist, which regulates angiogenesis in a coordinated fashion.

KM survival analysis unraveled that higher F3 and VEGF-A expression correlates with shorter RFS in breast cancer of the TNBC subtype and p53 mutant patients of the basal subtype, respectively. A subtype-specific impact on breast cancer survival was also noted for other constituents of the TF pathway, with F7 being associated with a better prognosis in the whole patient collective and the luminal A subtype, and F2R (PAR1)/F2RL1 (PAR2) acting as negative prognostic indicators particularly in the aggressive ER- and HER2 + subtypes. In agreement, breast cancer TF/F3 is an independent prognostic factor for overall survival and has a predictive value for distant metastasis [52]. Moreover, previous pilot studies have suggested that PAR1 is upregulated in aggressive breast cancer [53,54], and mechanistic studies have demonstrated that PAR1 induction promotes breast cancer progression and metastasis [55]. PAR2 is highly expressed in infiltrative ductal breast cancer tissue [56] and has been shown to be associated with distant disease-free survival in a previous study on 221 breast cancer patients [57]. It is well-established that VEGF is overexpressed in breast cancer tissue specimens, and high VEGF expression is coupled with poor prognostic factors, namely tumor size, TNM stage II and III, and lymph node metastasis, which affect the overall survival [58]. Connecting these findings, another study reported a significant link of F2RL1, F3, and VEGF-A expression in the clinical setting of primary breast cancer, where patients with phosphorylated F3 and F2RL1-expressed tumor tissue specimen are prone to recurrence. Co-expression of phosphorylated F3 and F2RL1 significantly correlates with shorter recurrence-free survival, while co-expression of F2RL1 and VEGF-A is linked with an aggressive phenotype [59]. Our study on over 3000 patients firmly establishes a prognostic role for the TF pathway in breast cancer and provides a novel link to Sdc-1.

The clinicopathological findings are further supported by work in vitro and in model systems: LX-2 hepatic stellate cells-expressing F2RL1 enhance tumor growth via angiogenesis in vivo using a xenograft of hepatocellular carcinoma model [60] and in an intestinal tumor model [61]. In a mouse model of mammary tumor virus-polyoma middle T (PyMT), F2RL1 (PAR2), but not F2R (PAR1), signaling accelerates angiogenesis and tumor growth [62]. These findings further underscore the clinical significance of Sdc-1 expression in regulating multiple interconnected factors (F3, F2RL1, and VEGF-A) involved in various hallmarks of cancers, angiogenesis, and tumor recurrence. This lines up well with our previous study, indicating the pivotal role of Sdc-1 in regulating cancer stem cells in IBC tumors [14]. Angiogenesis of prostate cancer was inhibited possibly via suppressor of cytokine signaling 6 (SOCS6) overexpression-mediated F7 downregulation [63]. Concordantly, our qPCR data analysis uncovered diminished F7 mRNA expression levels in both Sdc-1-silenced SUM-149 and MDA-MB-231 cell relative to control cells. In addition, F2RL1 mRNA levels were decreased in MDA-MB-231 cells upon Sdc-1 depletion. This may serve as a further explanation for alleviating angiogenesis in our experimental model. Interestingly, in retinal ganglion cells, F2RL1 translocates from the plasma membrane to the nucleus facilitating recruitment of the transcription factor Sp1, which in turn leads to induction of VEGF-A expression and subsequent neovascularization [64]. We and others have shown that Sp1 expression is influenced and regulated by Sdc-1 expression [23,65]. Accordingly, we propose a similar mechanism may function to regulate VEGF-A expression by Sdc-1 in our models. Taken together, it can be inferred that the identified panel of factors may represent a further plausible explanation for the suppressive effect of Sdc-1 expression interference on angiogenesis. F2R expression has been found to promote angiogenesis via thrombin-induced VEGF synthesis and secretion in prostate cancer [66]. Surprisingly, we found overexpression of F2R mRNA upon Sdc-1 suppression by siRNA treatment in MDA-MB-231 and MDA-MB-468 cells, but no change of F2R mRNA expression was observed in SUM-149 cells. However, the effect of F2R overexpression on VEGF-A was not observed in our experimental models, as VEGF-A secretion was diminished post-Sdc-1 depletion in SUM-149 cells, suggesting a context-dependent effect. Moreover, as we previously mentioned above, F2R expression is dispensable for mammary tumor growth and angiogenesis in the mammary tumor virus-polyoma middle T (PyMT) mouse model [62]. 

Apart from TF, ET-1 is one of the major angiogenic factors and correlates with VEGF expression in breast cancer [67,68]. Our qPCR and ELISA data analysis demonstrated that EDN1/ET-1 expression on mRNA levels and in secretome was reduced in Sdc-1-deficient SUM-149 and MDA-MB-468 cells. In accordance, downregulation of EDN1 expression dampened endothelial cell tube formation in human gastric cancer cells [69], implying that Sdc-1 may modulate angiogenesis via modulation of EDN1 expression. 

Further clues for angiogenesis regulation are IGFBP1 and IGFBP2. Our qPCR data show downregulation of IGFBP1 mRNA expression levels upon Sdc-1 knockdown in MDA-MB-231 and MDA-MB-468 cells, but IGFBP2 mRNA expression was only repressed in MDA-MB-468 cells. In addition, the secretome analysis revealed downregulation of IGFBP1 and IGFBP2 in MDA-MB-231 cocultures, and of IGFBP1 in MDA-MB-468 cocultures. IGFBP1 acts as a proangiogenic factor contributing to HCV-associated with hepatocellular carcinoma pathogenesis [70] and that elevated IGFBP1 expression is associated with hematogenous metastasis and poor outcome of gastric cancer [71]. Moreover, the nuclear IGFBP2 augments angiogenesis via increasing the promoter transcriptional activity of VEGF in cancer cells [72]. Together, this provides further explanation for impaired angiogenesis upon Sdc-1 expression ablation in cancer cells, which apparently acts in a cell context-dependent manner.

A few caveats are associated with this study. Regarding independent confirmation of the angiogenesis assay with Sdc-1 siRNA sequence #2, a reduction of angiogenesis was seen in SUM-149 and MDA-MB-231 cells; however, these data were only statistically significant in the case of SUM-149. In contrast, downregulation of TF secretion was significant in both models for siRNA #1 and siRNA #2. Regarding the impact of Sdc-1 on angiogenic factor expression, we have highlighted the TF pathway as a novel finding of this study. Nevertheless, additional factors of this work, such as VEGF-A, ET-1 and the IGF-pathway, can be expected to make an additional contribution to the Sdc-1-dependent angiogenesis phenotype, albeit in a cell type-specific manner. Cell type- and context-dependent effects for Sdc-1 are not surprising, as it acts as an important co-receptor for a multitude of signaling pathways, including receptor tyrosine kinases, G-protein-coupled chemokine receptors, morphogen signaling, and possible notch signaling [7,8,11]. Independent of the nature of the signaling pathways, it is remarkable that Sdc-1 depletion in all three TNBC cell lines resulted in a general decrease in the secretion of multiple proangiogenic factors (Figure 2). While we focused our in vitro investigation on the clinically highly relevant subtype of TNBC, our survival analysis demonstrated a prognostic impact of the TF pathway on different breast cancer subtypes, with F3 being of prognostic value only in p53 mutated TNBCs, barely missing significance (p = 0.06) in TNBC in general. This finding suggests that a further mechanistic evaluation of the role of Sdc-1 in connection to the TF pathway in other subtypes of breast cancer may be worthwhile. Our previous observation of an association of Sdc-1 with angiogenic factors in ductal carcinoma in situ of the breast is in line with this idea [16], as are our previous studies demonstrating altered signaling through the MAPK, Notch, Wnt, and IL-6-STAT3-pathways in Sdc-1-depleted cells as a mechanistic element of aberrant cytokine and angiogenic factor expression [9,14,23,73,74]. 

## 5. Conclusions

Our study establishes a novel role for TNBC cell-derived Sdc-1 in regulating components of the TF pathway and additional angiogenic factors, which promote angiogenesis in a 3D in vitro model. Notably, the Sdc-1-dependent factors have a prognostic value in breast cancer beyond TNBC, highlighting their clinicopathological relevance. Pharmacological targeting of Sdc-1, therefore, emerges as a potential therapeutic approach in TNBC and possibly additional subtypes of breast cancer, which does not only affect tumorigenic properties such as cell proliferation, invasive growth, and the cancer stem cell phenotype [14,73,74], but also tumor angiogenesis. The novel connection between Sdc-1 and the TF pathway may even trigger studies in non-oncological areas, including inflammation and thrombosis. Several preclinical approaches targeting Sdc-1 have already yielded positive results [75,76,77,78,79]. For example, application of the Sdc-1-targeting monoclonal antibody OC-46F2 curtails vascular maturation and tumor growth in mouse models of human melanoma and ovarian cancer [80]. In light of our present study findings, these data emphasize the potential therapeutic benefit of targeting cell-autonomous and tumor microenvironmental Sdc-1 against tumor angiogenesis of TNBC, which is worth exploring in future translational studies.

## Figures and Tables

**Table 1:** Prognostic value of coagulation factor III/TF expression in breast cancer patients stratified according to different clinicopathological categories.

Classification	Status	Cases	HR 95% CI	*p*-Value
ALL	-	3951	0.78 (0.7–0.87)	8.4 × 10^−5^
Estrogen receptor (ER)	Positive (+)	2061	0.75 (0.64–0.89)	0.00069
	Negative (−)	801	1.17 (0.93–1.46)	0.18
Progesterone receptor (PR)	Positive	589	0.62 (0.43–0.88)	0.0074
	Negative	549	1.06 (0.79–1.41)	0.71
HER2	Positive	252	1.32 (0.85–2.04)	0.22
	Negative	800	0.81 (0.62–1.05)	0.11
ER, PR, HER2	Negative	255	1.27 (0.83–1.95)	0.27
Intrinsic subtype	Luminal A	1933	0.74 (0.62–0.87)	0.00044
	Luminal B	1149	0.81 (0.67–0.98)	0.031
	HER2	251	1.14 (0.78–1.68)	0.49
	Basal	618	1.13 (0.88–1.45)	0.35
ER, PR, HER2	Negative	186	1.63 (0.97–2.72)	0.06
Intrinsic subtype	Basal			
ER, PR	Negative	115	0.89 (0.49–1.63)	0.72
HER2	Positive			
Lymph node	Positive	1133	0.86 (0.7–1.04)	0.12
	Negative	2020	0.9 (0.76–1.06)	0.22
Grade	1	345	0.85 (0.51–1.44)	0.55
	2	901	0.94 (0.74–1.19)	0.59
	3	903	1.02 (0.82–1.27)	0.86
p53	Mutated	188	1.85 (1.13–3.01)	0.012
	Wild type	273	0.72 (0.47–1.1)	0.13
p53 mutated	ER+	65	0.83 (0.39–1.77)	0.63
	ER−	66	1.4 (0.65–3)	0.38
	PR+	25	1.52 (0.43–5.39)	0.51
	PR−	50	0.62 (0.25–1.53)	0.3
	HER2+	23	0.63 (0.18–2.23)	0.47
	HER2−	55	0.72 (0.29–1.76)	0.47
	Basal	74	2.56 (1.05–6.23)	0.032
p53 wild type	ER+	234	0.83 (0.53–1.3)	0.43
	ER−	29	0.71 (0.19–2.69)	0.61
	PR+	52	0.39 (0.12–1.27)	0.11
	PR−	24	4.4 (0.91–21.26)	0.044
	HER2−	60	1.04 (0.43–2.5)	0.93

## Data Availability

All data are available within the article.

## Supplementary Materials

The following are available online at https://www.mdpi.com/article/10.3390/cancers13102318/s1, Figure S1: Sdc-1 depletion in TNBC cells restrains angiogenesis network formation of HUVECs and reduces the secretion of coagulation factor III/TF. Figure S2: Quantitative analysis of angiogenic factor secretion as determined by angiogenesis array. Figure S3. RNASeq expression data of angiogenic factors in 33 breast cancer cell lines as retrieved from the EMBO EBI expression atlas. Table S1: Primers sequences used in the study. Table S2. Summary of angiogenesis array proteome profiling results for conditioned media collected from control and Sdc-1 siRNA-treated SUM-149 cells cultured in 2D or 3D co-culture with HUVEC cells and analyzed by Proteome Profiler™ Human Angiogenesis Antibody Array. Table S3. KM Plotter analysis reveals the prognostic impact of VEGF-A expression in breast cancer patients stratified according to different clinicopathological categories. Table S4. Gene ontology (GO) analysis of Sdc-1, F3, F7, VEGF-A, EDN1, F2R, F2RL1, IGFBP1, and IGFBP2. Table S5. The biological processes associated with Sdc-1, F3, F7, VEGF-A, EDN1, F2R, F2RL1, IGFBP1, and IGFBP2 according to KEGG enrichment analysis.

## References

[1] Torre L.A., Bray F., Siegel R.L., Ferlay J., Lortet-Tieulent J., Jemal A. (2015). Global cancer statistics, 2012. CA Cancer J. Clin..

[2] Yersal O., Barutca S. (2014). Biological subtypes of breast cancer: Prognostic and therapeutic implications. World J. Clin. Oncol..

[3] De Palma M., Biziato D., Petrova T.V. (2017). Microenvironmental regulation of tumour angiogenesis. Nat. Rev. Cancer.

[4] Fragomeni S.M., Sciallis A., Jeruss J.S. (2018). Molecular Subtypes and Local-Regional Control of Breast Cancer. Surg. Oncol. Clin. N. Am..

[5] Hanahan D., Weinberg R.A. (2011). Hallmarks of cancer: The next generation. Cell.

[6] Zhu S., Kisiel W., Lu Y.J., Petersen L.C., Ndungu J.M., Moore T.W., Parker E.T., Sun A., Liotta D.C., El-Rayes B.F. (2014). Tumor angiogenesis therapy using targeted delivery of Paclitaxel to the vasculature of breast cancer metastases. J. Drug. Deliv..

[7] Onyeisi J.O.S., Ferreira B.Z.F., Nader H.B., Lopes C.C. (2020). Heparan sulfate proteoglycans as targets for cancer therapy: A review. Cancer Biol. Ther..

[8] Hassan N., Greve B., Espinoza-Sánchez N.A., Götte M. (2021). Cell-surface heparan sulfate proteoglycans as multifunctional integrators of signaling in cancer. Cell. Signal..

[9] Baston-Büst D.M., Götte M., Janni W., Krüssel J.S., Hess A.P. (2010). Syndecan-1 knock-down in decidualized human endometrial stromal cells leads to significant changes in cytokine and angiogenic factor expression patterns. Reprod. Biol. Endocrinol..

[10] Skandalis S.S., Dobra K., Götte M., Karousou E., Misra S. (2015). Impact of Extracellular Matrix on Cellular Behavior: A Source of Molecular Targets in Disease. Biomed. Res. Int..

[11] Karamanos N.K., Piperigkou Z., Theocharis A.D., Watanabe H., Franchi M., Baud S., Brézillon S., Götte M., Passi A., Vigetti D. (2018). Proteoglycan Chemical Diversity Drives Multifunctional Cell Regulation and Therapeutics. Chem. Rev..

[12] Hassan H., Greve B., Pavao M.S., Kiesel L., Ibrahim S.A., Götte M. (2013). Syndecan-1 modulates β-integrin-dependent and interleukin-6-dependent functions in breast cancer cell adhesion, migration, and resistance to irradiation. FEBS J..

[13] Szatmári T., Ötvös R., Hjerpe A., Dobra K. (2015). Syndecan-1 in Cancer: Implications for Cell Signaling, Differentiation, and Prognostication. Dis. Markers.

[14] Ibrahim S.A., Gadalla R., El-Ghonaimy E.A., Samir O., Mohamed H.T., Hassan H., Greve B., El-Shinawi M., Mohamed M.M., Götte M. (2017). Syndecan-1 is a novel molecular marker for triple negative inflammatory breast cancer and modulates the cancer stem cell phenotype via the IL-6/STAT3, Notch and EGFR signaling pathways. Mol. Cancer.

[15] Sayyad M.R., Puchalapalli M., Vergara N.G., Wangensteen S.M., Moore M., Mu L., Edwards C., Anderson A., Kall S., Sullivan M. (2019). Syndecan-1 facilitates breast cancer metastasis to the brain. Breast Cancer Res. Treat..

[16] Götte M., Kersting C., Radke I., Kiesel L., Wülfing P. (2007). An expression signature of syndecan-1 (CD138), E-cadherin and c-met is associated with factors of angiogenesis and lymphangiogenesis in ductal breast carcinoma in situ. Breast Cancer Res..

[17] Götte M., Joussen A.M., Klein C., Andre P., Wagner D.D., Hinkes M.T., Kirchhof B., Adamis A.P., Bernfield M. (2002). Role of syndecan-1 in leukocyte-endothelial interactions in the ocular vasculature. Investig. Ophthalmol. Vis. Sci..

[18] Elenius V., Götte M., Reizes O., Elenius K., Bernfield M. (2004). Inhibition by the soluble syndecan-1 ectodomains delays wound repair in mice overexpressing syndecan-1. J. Biol. Chem..

[19] Beauvais D.M., Ell B.J., McWhorter A.R., Rapraeger A.C. (2009). Syndecan-1 regulates alphavbeta3 and alphavbeta5 integrin activation during angiogenesis and is blocked by synstatin, a novel peptide inhibitor. J. Exp. Med..

[20] Jing Z., Wei-Jie Y., Yi-Feng Z.G., Jing H. (2016). Downregulation of Syndecan-1 induce glomerular endothelial cell dysfunction through modulating internalization of VEGFR-2. Cell. Signal..

[21] Maeda T., Desouky J., Friedl A. (2006). Syndecan-1 expression by stromal fibroblasts promotes breast carcinoma growth in vivo and stimulates tumor angiogenesis. Oncogene.

[22] Brand C., Greve B., Bölling T., Eich H.T., Willich N., Harrach S., Hintelmann H., Lenz G., Mesters R.M., Kessler T. (2020). Radiation synergizes with antitumor activity of CD13-targeted tissue factor in a HT1080 xenograft model of human soft tissue sarcoma. PLoS ONE.

[23] Ibrahim S.A., Yip G.W., Stock C., Pan J.W., Neubauer C., Poeter M., Pupjalis D., Koo C.Y., Kelsch R., Schüle R. (2012). Targeting of syndecan-1 by microRNA miR-10b promotes breast cancer cell motility and invasiveness via a Rho-GTPase- and E-cadherin-dependent mechanism. Int. J. Cancer.

[24] Carpentier G., Berndt S., Ferratge S., Rasband W., Cuendet M., Uzan G., Albanese P. (2020). Angiogenesis Analyzer for ImageJ - A comparative morphometric analysis of “Endothelial Tube Formation Assay” and “Fibrin Bead Assay”. Sci. Rep..

[25] Györffy B., Lanczky A., Eklund A.C., Denkert C., Budczies J., Li Q., Szallasi Z. (2010). An online survival analysis tool to rapidly assess the effect of 22,277 genes on breast cancer prognosis using microarray data of 1,809 patients. Breast Cancer Res. Treat..

[26] Szklarczyk D., Gable A.L., Lyon D., Junge A., Wyder S., Huerta-Cepas J., Simonovic M., Doncheva N.T., Morris J.H., Bork P. (2019). STRING v11: Protein-protein association networks with increased coverage, supporting functional discovery in genome-wide experimental datasets. Nucleic Acids Res..

[27] Gong I.Y., Fox N.S., Huang V., Boutros P.C. (2018). Prediction of early breast cancer patient survival using ensembles of hypoxia signatures. PLoS ONE.

[28] Ibrahim S.A., Hassan H., Götte M. (2014). MicroRNA regulation of proteoglycan function in cancer. FEBS J..

[29] Hassan N., Rutsch N., Győrffy B., Espinoza-Sánchez N.A., Götte M. (2020). SETD3 acts as a prognostic marker in breast cancer patients and modulates the viability and invasion of breast cancer cells. Sci. Rep..

[30] Toi M., Hoshina S., Takayanagi T., Tominaga T. (1994). Association of vascular endothelial growth factor expression with tumor angiogenesis and with early relapse in primary breast cancer. Jpn. J. Cancer Res..

[31] Delli Carpini J., Karam A.K., Montgomery L. (2010). Vascular endothelial growth factor and its relationship to the prognosis and treatment of breast, ovarian, and cervical cancer. Angiogenesis.

[32] Petryszak R., Fonseca N.A., Füllgrabe A., Huerta L., Keays M., Tang Y.A., Brazma A. (2017). The RNASeq-er API-a gateway to systematically updated analysis of public RNA-seq data. Bioinformatics.

[33] Cole M., Bromberg M. (2013). Tissue factor as a novel target for treatment of breast cancer. Oncologist.

[34] Bernfield M., Kokenyesi R., Kato M., Hinkes M.T., Spring J., Gallo R.L., Lose E.J. (1992). Biology of the syndecans: A family of transmembrane heparan sulfate proteoglycans. Annu. Rev. Cell Biol..

[35] Rapraeger A.C. (2013). Synstatin: A selective inhibitor of the syndecan-1-coupled IGF1R-αvβ3 integrin complex in tumorigenesis and angiogenesis. FEBS J..

[36] Teixeira F., Götte M. (2020). Involvement of Syndecan-1 and Heparanase in Cancer and Inflammation. Adv. Exp. Med. Biol..

[37] Kuczynski E.A., Vermeulen P.B., Pezzella F., Kerbel R.S., Reynolds A.R. (2019). Vessel co-option in cancer. Nat. Rev. Clin. Oncol..

[38] Khotskaya Y.B., Dai Y., Ritchie J.P., MacLeod V., Yang Y., Zinn K., Sanderson R.D. (2009). Syndecan-1 is required for robust growth, vascularization, and metastasis of myeloma tumors in vivo. J. Biol. Chem..

[39] Lamorte S., Ferrero S., Aschero S., Monitillo L., Bussolati B., Omedè P., Ladetto M., Camussi G. (2012). Syndecan-1 promotes the angiogenic phenotype of multiple myeloma endothelial cells. Leukemia.

[40] Abdollahi A., Hahnfeldt P., Maercker C., Gröne H.J., Debus J., Ansorge W., Folkman J., Hlatky L., Huber P.E. (2004). Endostatin’s antiangiogenic signaling network. Mol. Cell.

[41] D’Asti E., Kool M., Pfister S.M., Rak J. (2014). Coagulation and angiogenic gene expression profiles are defined by molecular subgroups of medulloblastoma: Evidence for growth factor-thrombin cross-talk. J. Thromb. Haemost..

[42] Ruf W., Yokota N., Schaffner F. (2010). Tissue factor in cancer progression and angiogenesis. Thromb. Res..

[43] Serra R., Buffone G., Montemurro R., de Franciscis S. (2013). Axillary vein thrombosis as the first clinical manifestation of inflammatory breast cancer: Report of a case. Surg. Today.

[44] Nadir Y., Brenner B., Zetser A., Ilan N., Shafat I., Zcharia E., Goldshmidt O., Vlodavsky I. (2006). Heparanase induces tissue factor expression in vascular endothelial and cancer cells. J. Thromb. Haemost..

[45] Chen L., Sanderson R.D. (2009). Heparanase regulates levels of syndecan-1 in the nucleus. PLoS ONE.

[46] El-Nadi M., Hassan H., Saleh M.E., Nassar E., Ismail Y.M., Amer M., Greve B., Götte M., El-Shinawi M., Ibrahim S.A. (2020). Induction of heparanase via IL-10 correlates with a high infiltration of CD163+ M2-type tumor-associated macrophages in inflammatory breast carcinomas. Matrix Biol. Plus.

[47] Peña E., de la Torre R., Arderiu G., Slevin M., Badimon L. (2017). mCRP triggers angiogenesis by inducing F3 transcription and TF signalling in microvascular endothelial cells. Thromb. Haemost..

[48] Huang R., Andersen L.M.K., Rofstad E.K. (2017). Metastatic pathway and the microvascular and physicochemical microenvironments of human melanoma xenografts. J. Transl. Med..

[49] van den Berg Y.W., van den Hengel L.G., Myers H.R., Ayachi O., Jordanova E., Ruf W., Spek C.A., Reitsma P.H., Bogdanov V.Y., Versteeg H.H. (2009). Alternatively spliced tissue factor induces angiogenesis through integrin ligation. Proc. Natl. Acad. Sci. USA.

[50] van den Berg Y.W., Osanto S., Reitsma P.H., Versteeg H.H. (2012). The relationship between tissue factor and cancer progression: Insights from bench and bedside. Blood.

[51] Han X., Guo B., Li Y., Zhu B. (2014). Tissue factor in tumor microenvironment: A systematic review. J. Hematol. Oncol..

[52] Ueno T., Toi M., Koike M., Nakamura S., Tominaga T. (2000). Tissue factor expression in breast cancer tissues: Its correlation with prognosis and plasma concentration. Br. J. Cancer.

[53] Tiburcio M., Costa S.M., Duarte M.d.F., Schmitt F.C., Filho A.L. (2012). Characterization of PAR1 and FGFR1 expression in invasive breast carcinomas: Prognostic significance. Oncol. Lett..

[54] Hernández N.A., Correa E., Avila E.P., Vela T.A., Pérez V.M. (2009). PAR1 is selectively over expressed in high grade breast cancer patients: A cohort study. J. Transl. Med..

[55] Wang Y., Liao R., Chen X., Ying X., Chen G., Li M., Dong C. (2020). Twist-mediated PAR1 induction is required for breast cancer progression and metastasis by inhibiting Hippo pathway. Cell Death Dis..

[56] Matej R., Mandáková P., Netíková I., Poucková P., Olejár T. (2007). Proteinase-activated receptor-2 expression in breast cancer and the role of trypsin on growth and metabolism of breast cancer cell line MDA MB-231. Physiol. Res..

[57] Lidfeldt J., Bendahl P.O., Forsare C., Malmström P., Fernö M., Belting M. (2015). Protease Activated Receptors 1 and 2 Correlate Differently with Breast Cancer Aggressiveness Depending on Tumor ER Status. PLoS ONE.

[58] Li S., Wang L., Meng Y., Chang Y., Xu J., Zhang Q. (2017). Increased levels of LAPTM4B, VEGF and survivin are correlated with tumor progression and poor prognosis in breast cancer patients. Oncotarget.

[59] Rydén L., Grabau D., Schaffner F., Jönsson P.E., Ruf W., Belting M. (2010). Evidence for tissue factor phosphorylation and its correlation with protease-activated receptor expression and the prognosis of primary breast cancer. Int. J. Cancer.

[60] Mußbach F., Ungefroren H., Günther B., Katenkamp K., Henklein P., Westermann M., Settmacher U., Lenk L., Sebens S., Müller J.P. (2016). Proteinase-activated receptor 2 (PAR2) in hepatic stellate cells—Evidence for a role in hepatocellular carcinoma growth in vivo. Mol. Cancer.

[61] Kawaguchi M., Yamamoto K., Kataoka H., Izumi A., Yamashita F., Kiwaki T., Nishida T., Camerer E., Fukushima T. (2020). Protease-activated receptor-2 accelerates intestinal tumor formation through activation of nuclear factor-κB signaling and tumor angiogenesis in Apc(Min/+) mice. Cancer Sci..

[62] Versteeg H.H., Schaffner F., Kerver M., Ellies L.G., Andrade-Gordon P., Mueller B.M., Ruf W. (2008). Protease-activated receptor (PAR) 2, but not PAR1, signaling promotes the development of mammary adenocarcinoma in polyoma middle T mice. Cancer Res..

[63] Yuan D., Wang W., Su J., Zhang Y., Luan B., Rao H., Cheng T., Zhang W., Xiao S., Zhang M. (2018). SOCS6 Functions as a Tumor Suppressor by Inducing Apoptosis and Inhibiting Angiogenesis in Human Prostate Cancer. Curr. Cancer Drug Targets.

[64] Joyal J.S., Nim S., Zhu T., Sitaras N., Rivera J.C., Shao Z., Sapieha P., Hamel D., Sanchez M., Zaniolo K. (2014). Subcellular localization of coagulation factor II receptor-like 1 in neurons governs angiogenesis. Nat. Med..

[65] Szatmári T., Mundt F., Kumar-Singh A., Möbus L., Ötvös R., Hjerpe A., Dobra K. (2017). Molecular targets and signaling pathways regulated by nuclear translocation of syndecan-1. BMC Cell Biol..

[66] Latil A., Bièche I., Chêne L., Laurendeau I., Berthon P., Cussenot O., Vidaud M. (2003). Gene expression profiling in clinically localized prostate cancer: A four-gene expression model predicts clinical behavior. Clin. Cancer Res..

[67] Wülfing P., Kersting C., Tio J., Fischer R.J., Wülfing C., Poremba C., Diallo R., Böcker W., Kiesel L. (2004). Endothelin-1-, endothelin-A-, and endothelin-B-receptor expression is correlated with vascular endothelial growth factor expression and angiogenesis in breast cancer. Clin. Cancer Res..

[68] Fischgräbe J., Götte M., Michels K., Kiesel L., Wülfing P. (2010). Targeting endothelin A receptor enhances anti-proliferative and anti-invasive effects of the HER2 antibody trastuzumab in HER2-overexpressing breast cancer cells. Int. J. Cancer.

[69] Xie M., Dart D.A., Guo T., Xing X.F., Cheng X.J., Du H., Jiang W.G., Wen X.Z., Ji J.F. (2018). MicroRNA-1 acts as a tumor suppressor microRNA by inhibiting angiogenesis-related growth factors in human gastric cancer. Gastric. Cancer.

[70] Benkheil M., Paeshuyse J., Neyts J., Van Haele M., Roskams T., Liekens S. (2018). HCV-induced EGFR-ERK signaling promotes a pro-inflammatory and pro-angiogenic signature contributing to liver cancer pathogenesis. Biochem. Pharmacol..

[71] Sato Y., Inokuchi M., Takagi Y., Kojima K. (2019). IGFBP1 Is a Predictive Factor for Haematogenous Metastasis in Patients With Gastric Cancer. Anticancer Res..

[72] Azar W.J., Zivkovic S., Werther G.A., Russo V.C. (2014). IGFBP-2 nuclear translocation is mediated by a functional NLS sequence and is essential for its pro-tumorigenic actions in cancer cells. Oncogene.

[73] Ibrahim S.A., Hassan H., Vilardo L., Kumar S.K., Kumar A.V., Kelsch R., Schneider C., Kiesel L., Eich H.T., Zucchi I. (2013). Syndecan-1 (CD138) modulates triple-negative breast cancer stem cell properties via regulation of LRP-6 and IL-6-mediated STAT3 signaling. PLoS ONE.

[74] Nikolova V., Koo C.Y., Ibrahim S.A., Wang Z., Spillmann D., Dreier R., Kelsch R., Fischgräbe J., Smollich M., Rossi L.H. (2009). Differential roles for membrane-bound and soluble syndecan-1 (CD138) in breast cancer progression. Carcinogenesis.

[75] Tassone P., Goldmacher V.S., Neri P., Gozzini A., Shammas M.A., Whiteman K.R., Hylander-Gans L.L., Carrasco D.R., Hideshima T., Shringarpure R. (2004). Cytotoxic activity of the maytansinoid immunoconjugate B-B4-DM1 against CD138+ multiple myeloma cells. Blood.

[76] von Strandmann E.P., Hansen H.P., Reiners K.S., Schnell R., Borchmann P., Merkert S., Simhadri V.R., Draube A., Reiser M., Purr I. (2006). A novel bispecific protein (ULBP2-BB4) targeting the NKG2D receptor on natural killer (NK) cells and CD138 activates NK cells and has potent antitumor activity against human multiple myeloma in vitro and in vivo. Blood.

[77] Ikeda H., Hideshima T., Fulciniti M., Lutz R.J., Yasui H., Okawa Y., Kiziltepe T., Vallet S., Pozzi S., Santo L. (2009). The monoclonal antibody nBT062 conjugated to cytotoxic Maytansinoids has selective cytotoxicity against CD138-positive multiple myeloma cells in vitro and in vivo. Clin. Cancer Res..

[78] Rousseau C., Ruellan A.L., Bernardeau K., Kraeber-Bodéré F., Gouard S., Loussouarn D., Saï-Maurel C., Faivre-Chauvet A., Wijdenes J., Barbet J. (2011). Syndecan-1 antigen, a promising new target for triple-negative breast cancer immuno-PET and radioimmunotherapy. A preclinical study on MDA-MB-468 xenograft tumors. EJNMMI Res..

[79] Espinoza-Sánchez N.A., Götte M. (2020). Role of cell surface proteoglycans in cancer immunotherapy. Semin. Cancer Biol..

[80] Orecchia P., Conte R., Balza E., Petretto A., Mauri P., Mingari M.C., Carnemolla B. (2013). A novel human anti-syndecan-1 antibody inhibits vascular maturation and tumour growth in melanoma. Eur. J. Cancer.

