# Supplementary material for: Syndecan-1 Promotes Angiogenesis in Triple-Negative Breast Cancer through the Prognostically Relevant Tissue Factor Pathway and Additional Angiogenic Routes
Source: Cancers (Basel). 2021 May 12;13(10):2318. doi: 10.3390/cancers13102318 (PMC8150756; doi:10.3390/cancers13102318)
Supplement: Supplementary file 1 [file cancers-13-02318-s001.zip › cancers-1211352-supplementary.pdf]

Supplementary Tables

Table S1. Primers sequences used in the study.

**Taq Man Assays**	
**Gene name**	**Code**
β-Actin	Hs 99999903 m1
EDN1	Hs 00174961 m1
IGFBP1	Hs 00236877_m1
IGFBP2	Hs 00167151_m1
VEGFA	Hs 00900054_m1
**SYBR green primers**	
**Gene**	Primer sequences
β-Actin	Forward: 5′-CAA AGA CCT GTA CGC CAA CAC-3′<br>Reverse: 5′-CAT ACT CCT GCT TGC TGA TCC-3′
Sdc-1	Forward: 5′-AGG ACG AAG GCA GCT ACT CCT-3′<br>Reverse: 5′-TTT GGT GGG CTT CTG GTA GG-3′
F3	Forward: 5′-CAG AGT TCA CAC CTT ACC TGG AG-3′<br>Reverse: 5′-GTT GTT CCT TCT GAC TAA AGT CCG-3′
F7	Forward: 5′-CCT CAA GTC CAT GCC AGA ATG G-3′<br>Reverse: 5′-CAC AGA TCA GCT GGT CAT CCT TG-3′
F2R	Forward: 5′-GTT TCT GGC TGT GGT GTA TCC C-3′<br>Reverse: 5′-CCT GGA TGG TTT GCT CCT TGA G-3′
F2RL1	Forward: 5′-CTC CTC TCT GTC ATC TGG TTC C-3′<br>Reverse: 5′-TGC ACA CTG AGG CAG GTC ATG A-3′

Table S2. Summary of angiogenesis array proteome profiling results for conditioned media collected from control and Sdc-1 siRNA treated SUM-149 cells cultured in 2D or 3D coculture with HUVEC cells and analysed by Proteome Profiler™ Human Angiogenesis Antibody Array. See also Figure 2A in the main manuscript.

Gene product	ID	Co-culture Ctrl siRNA	Co-culture Sdc-1 siRNA	SUM-149 Ctrl siRNA	SUM-149 Sdc-1 siRNA
**Proteins affected by Sdc-1 siRNA**					
Angiogenin (ANG)	283	+	+/-	+	-
Coagulation Factor III (TF/F3)	2152	+	+/-	+++	++
Endothelin-1 (EDN1)	1906	+	+	++	+
IGFBP1	3484	+/-	-	-	-
IGFBP2	3485	+	+	+++	++
IGFBP3	3486	-	-	+/-	-
IL-8 (CXCL8)	3576	++	++	+++	++
Platelet Factor 4 (CXCL4)	5196	++	++	++	+
uPA	5328	++	++	+	-
VEGF	7422	+	+/-	+++	++
**Proteins affected by co-culture**					
Angiopoietin-2 (Ang-2)	285	++	++	-	-
Amphiregulin (AR)	374	++	++	+++	+++
CXCL16	58191	++	++	+++	+++
DPPIV (CD26)	1803	+	+	-	-
EG-VEGF (PK1)	84432	+	+	-	-
Endoglin (CD105)	2022	+	+	-	-
MMP-9	4318	+/-	+/-	-	-
Pentraxin 3 (TSG14)	5806	++	++	+++	+++
PDGF-AA	5154	+/-	+/-	-	-
PlGF	5228	++	++	-	-
Serpin E1 (PAI-1)	5054	+++	+++	-	-
**Additional Proteins**					
TIMP-1 and Thrombospondin-1 were highly expressed (+++) irrespective of the culture or siRNA treatment conditions. The following proteins were not detectable, neither in the coculture nor in the SUM-149 culture: Activin A, ADAMTS-1, Angiopoietin-1, Angiostatin/Plasminogen, Endostatin/Collagen XVIII, Artemin, EGF, FGF acidic, FGF basic, FGF-4, FGF-7, GDNF, GM-CSF, HB-EGF, HGF, IL-1β, LAP (TGF-β1), Leptin, MCP-1, MIP-1α, MMP-8, NRG1-β1, PD-ECGF, PDGF-AB/PDGF-BB, Persephin, Prolactin, Serpin B5, Serpin F1, TIMP-4, Thrombospondin-2, Vasohibin and VEGF-C.					

***=very high expression, ** high/regular expression, + low expression, +/- barely detectable, - not detectable. Ctrl = Control.

Table S3. KM Plotter analysis reveals the prognostic impact of VEGFA expression in breast cancer patients stratified according to different clinicopathological categories. See main manuscript for details.

Classification	Status	Cases	HR 95% CI	P-value
**ALL**	-	3951	1.34 (1.2 – 1.49)	1.8e-07
**Estrogen receptor (ER)**	Positive (+)	2061	1.03 (0.87 – 1.21)	0.76
	Negative (-)	801	1.45 (1.15 – 1.82)	0.0014
**Progesterone receptor (PR)**	Positive	589	1.48 (1.04 – 2.11)	0.028
	Negative	549	1.57 (1.17 – 2.11)	0.0025
**Her2**	Positive	252	2.35 (1.49 – 3.71)	0.00061
	Negative	800	1.73 (1.32 – 2.26)	4.8e-05
**ER, PR, Her2**	Negative	255	1.76 (1.14 – 2.73)	0.0095
**Intrinsic subtype**	Luminal A	1933	1.04 (0.88 – 1.23)	0.67
	Luminal B	1149	1.21 (1 – 1.47)	0.046
	Her2	251	1.84 (1.24 – 2.71)	0.002
	Basal	618	1.29 (1 – 1.66)	0.046
**ER, PR, Her2**	Negative	186	1.52 (0.91 – 2.54)	0.11
**Intrinsic subtype**	Basal			
**ER, PR**	Negative	115	1.71 (0.93 – 3.14)	0.078
**Her2**	Positive			
**Lymph node**	Positive	1133	1.39 (1.14 – 1.69)	0.001
	Negative	2020	1.19 (1.01 – 1.41)	0.043
**Grade**	1	345	0.88 (0.52 – 1.48)	0.63
	2	901	1.47 (1.15 – 1.87)	0.0018
	3	903	1.21 (0.97 – 1.5)	0.092
**p53**	Mutated	188	0.99 (0.61 – 1.59)	0.96
	Wild type	273	1.09 (0.72 – 1.67)	0.68
**p53 mutated**	ER+	65	2.01 (0.92 – 4.42)	0.076
	ER-	66	1.61 (0.75 – 3.48)	0.22
	PR+	25	0.77 (0.22 – 2.65)	0.67
	PR-	50	2.36 (0.94 – 5.94)	0.059
	Her2+	23	2.01 (0.56 – 7.2)	0.27
	Her2-	48	1.37 (0.57 – 3.3)	0.49
	Basal	74	1.32 (0.58 – 3)	0.51
**p53 wild type**	ER+	234	1.07 (0.68 – 1.67)	0.77
	ER-	29	1.27 (0.33 – 4.83)	0.73
	PR+	52	2.11 (0.68 – 6.51)	0.18
	PR-	24	1.06 (0.23 – 3.95)	0.93
	Her2-	60	1.24 (0.51 – 3)	0.63

Table S4. Gene ontology (GO) analysis of Sdc-1, F3, F7, VEGFA, EDN1, F2R, F2RL1, IGFBP1 and IGFBP2. The program STRING was used for this analysis.

Category	Pathway description	Observed gene count	False discovery rate	Matching proteins in your network (labels)
Biological process	GO:0030335 - positive regulation of cell migration	9	6.02E-09	EDN1,F2R,F2RL1,F3,F7,FLT1,IGF1,KDR,VEGFA
	GO:0050927 - positive regulation of positive chemotaxis	5	6.02E-09	F2RL1,F3,F7,KDR,VEGFA
	GO:0050921 - positive regulation of chemotaxis	6	8.27E-08	EDN1,F2RL1,F3,F7,KDR,VEGFA
	GO:0003018 - vascular process in circulatory system	6	1.60E-07	EDN1,EDNRA,EDNRB,F2R,F2RL1,VEGFA
	GO:0014068 - positive regulation of phosphatidylinositol 3-kinase signaling	5	1.60E-07	F2R,F2RL1,FLT1,IGF1,KDR
	GO:0008284 - positive regulation of cell population proliferation	9	1.70E-07	EDN1,EDNRB,F2R,F3,FLT1,IGF1,IGFBP2,KDR,VEGFA
	GO:0032879 - regulation of localization	12	1.70E-07	EDN1,EDNRA,EDNRB,F2R,F2RL1,F3,F7,FLT1,IGF1,KDR,SDC1,VEGFA
	GO:0030193 - regulation of blood coagulation	5	2.24E-07	EDN1,F2R,F2RL1,F3,F7
	GO:1902533 - positive regulation of intracellular signal transduction	9	2.32E-07	EDN1,F2R,F2RL1,F3,F7,FLT1,IGF1,KDR,VEGFA
	GO:0010033 - response to organic substance	12	5.14E-05	EDNRB,F2RL1,FLT1,KDR,SDC1,VEGFA
Molecular Function	GO:0005102 - Signaling receptor binding	10	6.37E-07	EDN1,EDNRB,F2R,F2RL1,F7,IGF1,IGFBP1,IGFBP2,KDR,VEGFA
	GO:0004962 - Endothelin receptor activity	2	9.52E-05	EDNRA,EDNRB
	GO:0008528 - G protein-coupled peptide receptor activity	4	9.52E-05	EDNRA,EDNRB,F2R,F2RL1
	GO:0019838 - Growth factor binding	4	9.52E-05	FLT1,IGFBP1,IGFBP2,KDR
	GO:0005515 - Protein binding	13	0.00013	EDN1,EDNRB,F2R,F2RL1,F3,F7,FLT1,IGF1,IGFBP1,IGFBP2,KDR,SDC1,VEGFA
	GO:0004888 - Transmembrane signaling receptor activity	7	0.00014	EDNRA,EDNRB,F2R,F2RL1,F3,FLT1,KDR
	GO:0015057 - Thrombin-activated receptor activity	2	0.00014	F2R,F2RL1
	GO:0005021 - Vascular endothelial growth factor-activated receptor activity	2	0.0002	FLT1,KDR
	GO:0031995 - Insulin-like growth factor II binding	2	0.00023	IGFBP1,IGFBP2
	GO:0031681 - G-protein beta-subunit binding	2	0.00024	F2R,F2RL1
Cellular Component	GO:0005615 - Extracellular space	8	3.18E-05	EDN1,F3,F7,FLT1,IGF1,IGFBP1,IGFBP2,VEGFA
	GO:0005576 - Extracellular region	10	4.08E-05	EDN1,F2R,F3,F7,FLT1,IGF1,IGFBP1,IGFBP2,KDR,VEGFA
	GO:0044459 - Plasma membrane part	10	4.59E-05	EDNRA,EDNRB,F2R,F2RL1,F3,FLT1,IGF1,IGFBP2,KDR,SDC1
	GO:0031226 - Intrinsic component of plasma membrane	8	0.0001	EDNRA,EDNRB,F2R,F2RL1,F3,FLT1,KDR,SDC1
	GO:0031982 - Vesicle	9	0.0001	EDN1,F2R,F2RL1,F7,FLT1,IGF1,IGFBP2,KDR,VEGFA
	GO:1905286 - Serine-type peptidase complex	2	0.0001	F3,F7
	GO:0012505 - Endomembrane system	11	0.00017	EDN1,EDNRB,F2R,F2RL1,F7,FLT1,IGF1,IGFBP1,KDR,SDC1,VEGFA
	GO:0005887 - Integral component of plasma membrane	7	0.00054	EDNRA,EDNRB,F2R,F2RL1,FLT1,KDR,SDC1
	GO:0031410 - Cytoplasmic vesicle	8	0.00054	EDN1,F2R,F2RL1,FLT1,IGF1,IGFBP2,KDR,VEGFA
	GO:0005886 - Plasma membrane	11	0.00064	EDNRA,EDNRB,F2R,F2RL1,F3,F7,FLT1,IGF1,IGFBP2,KDR,SDC1

Table S5. The biological processes associated with Sdc-1, F3, F7, VEGFA, EDN1, F2R, F2RL1, IGFBP1 and IGFBP2 according to KEGG enrichment analysis. The program STRING was used for this analysis.

Pathway description	Observed gene count	False discovery rate	Matching proteins in your network (labels)
hsa04015 - Rap1 signaling pathway	5	1.38E-05	F2R,FLT1,IGF1,KDR,VEGFA
hsa04066 - HIF-1 signaling pathway	4	1.94E-05	EDN1,FLT1,IGF1,VEGFA
hsa05418 - Fluid shear stress and atherosclerosis	4	4.20E-05	EDN1,KDR,SDC1,VEGFA
hsa04151 - PI3K-Akt signaling pathway	5	4.70E-05	F2R,FLT1,IGF1,KDR,VEGFA
hsa04510 - Focal adhesion	4	0.00011	FLT1,IGF1,KDR,VEGFA
hsa05205 - Proteoglycans in cancer	4	0.00011	IGF1,KDR,SDC1,VEGFA
hsa01521 - EGFR tyrosine kinase inhibitor resistance	3	0.00015	IGF1,KDR,VEGFA
hsa04014 - Ras signaling pathway	4	0.00015	FLT1,IGF1,KDR,VEGFA
hsa04610 - Complement and coagulation cascades	3	0.00015	F2R,F3,F7
hsa05200 - Pathways in cancer	5	0.00015	EDNRA,EDNRB,F2R,IGF1,VEGFA

Supplementary Figures

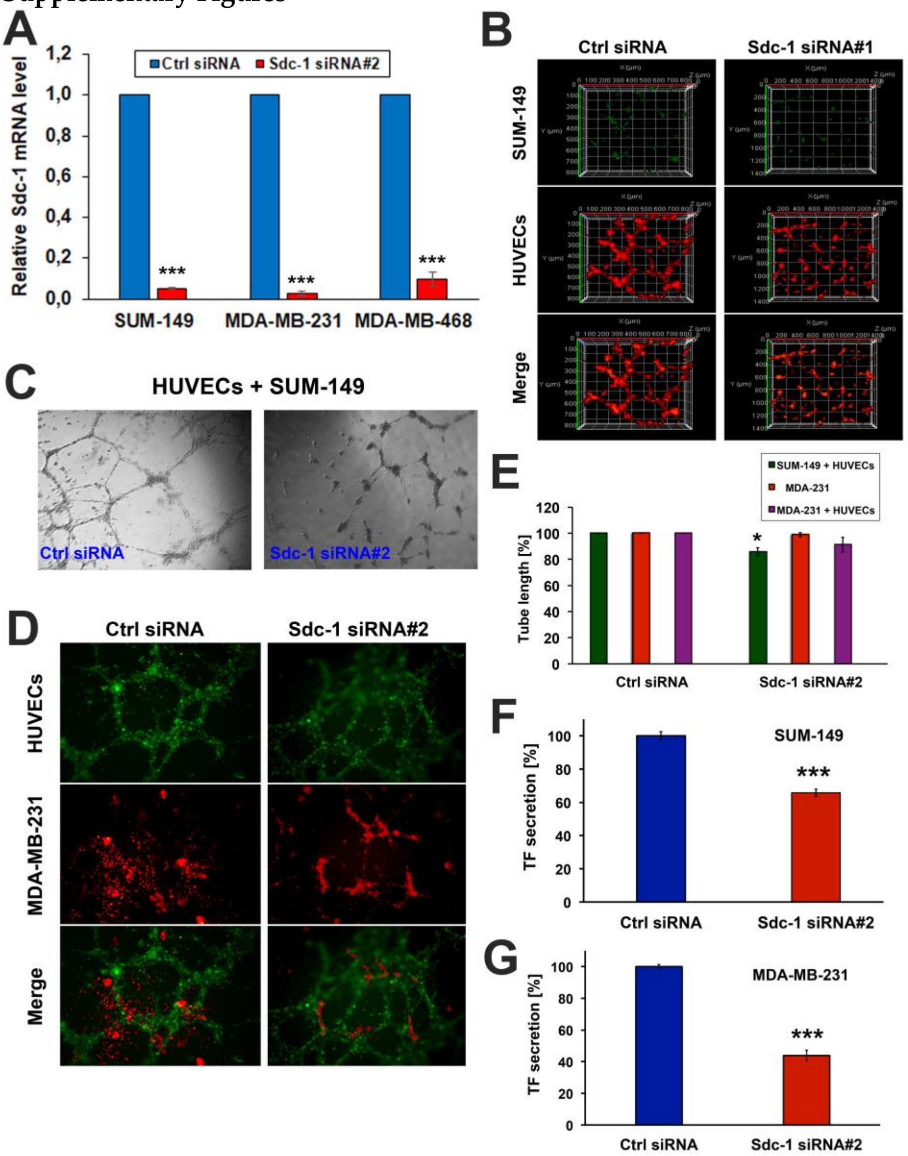

Figure S1: Sdc-1 depletion in TNBC cells restrains angiogenesis network formation of HUVECs and reduces the secretion of coagulation factor III/TF.

Sdc-1 depletion in TNBC cells restrains angiogenesis network formation of HUVECs and reduces the secretion of coagulation factor III/TF. (**A**) qPCR confirmation of successful Sdc-1 silencing using siRNA#2. Data represent the mean ± SEM, n ≥ 3. # $p < 0.001$ as determined by Student's *t*-test. (**B**–**E**) Sdc-1 knockdown in TNBC cells affects tubule network formation of HUVECs. (**B**) Confocal immunofluorescence microscopy of HUVECs (red fluorescent staining) co-cultured with control and Sdc-1-suppressed SUM-149 cells (green fluorescent staining) using Sdc-1 siRNA#1. (**C**) Phase-contrast images of HUVECs co-cultured with control and Sdc-1-suppressed SUM-149 cells using Sdc-1 siRNA#2. (**D**) Confocal immunofluorescence microscopy of HUVECs (green fluorescent staining) co-cultured with control and Sdc-1-suppressed MDA-MB-231 cells (red fluorescent staining) using Sdc-1 siRNA#2. (**E**) Quantitative analysis of HUVEC tubulogenesis, namely the total length of HUVEC tubules. Data represent the mean ± SEM, n = 3. * $p < 0.05$ as determined by Student's *t*-test. (**F**,**G**) Sdc-1 depletion in TNBC cells mediated by Sdc-1 siRNA#2 reduces the secretion of coagulation factor III/TF. Coagulation factor III/TF was quantified by ELISA in the cell culture supernatants of control and Sdc-1 knockdown SUM-149 (**F**) and MDA-MB-231 cells (**G**). MDA-MB-468 cells were not evaluated.

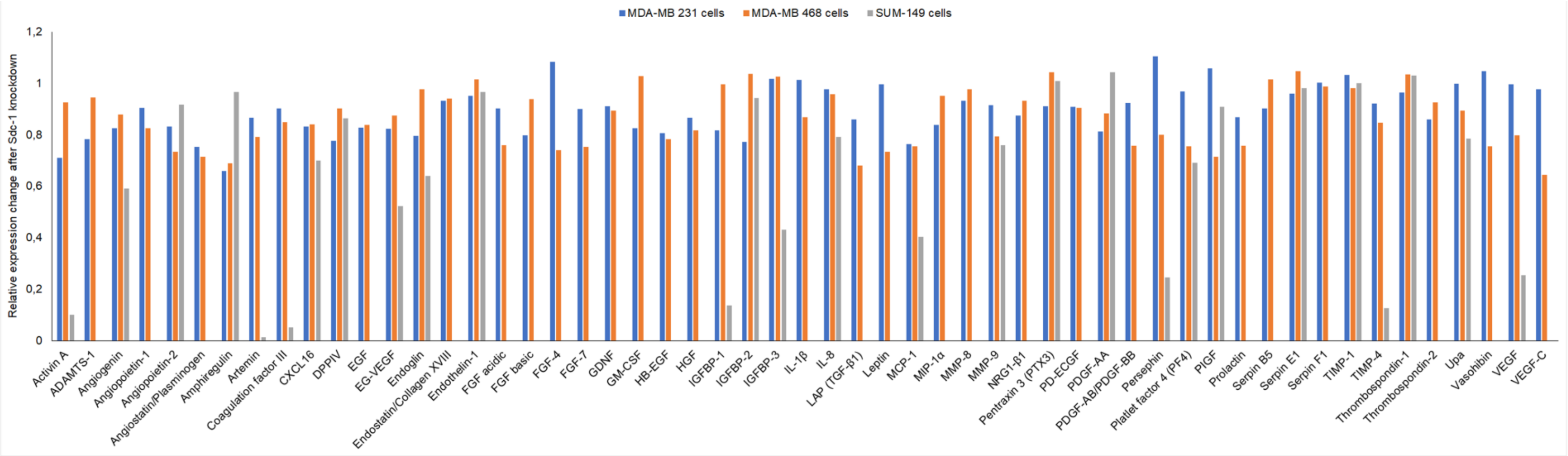

Figure S2: Quantitative analysis of angiogenic factor secretion as determined by angiogenesis array

Quantitative analysis of angiogenic factor secretion as determined by angiogenesis array. The conditioned media of Sdc-1-depleted SUM-149, MDA-MB-231, and MDA-MMB-468 cells in 48 h HUVEC coculture were analyzed by angiogenesis array (Figure 2 in main manuscript) and the results were quantified by densitometric scanning using NIH Image J software.

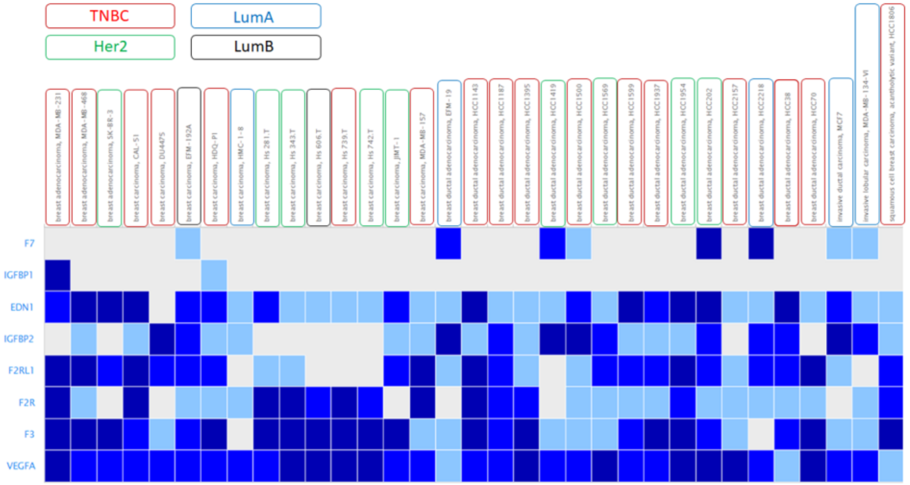

Figure S3: RNASeq expression data of angiogenic factors in 33 breast cancer cell lines as retrieved from the EMBO EBI expression atlas

RNASeq expression data of angiogenic factors in 33 breast cancer cell lines as retrieved from the EMBO EBI expression atlas, release 37, March 2021 [32]. Blue color indicates level of gene expression as FPKM (fragments per kilobase of exon model per million reads mapped), with light blue indicating values <5 and dark blue indicating expression >5 FKPM. Grey values indicate no detectable expression/no data. TNBC = triple-negative breast cancer, Her2 = Her2 subtype, LumA = luminal A, lumB = luminal B. The following are available online at www.mdpi.com/xxx/s1.
